# Design of programmable post-translational switch control platform for on-demand protein secretion in mammalian cells

**DOI:** 10.1093/nar/gkac916

**Published:** 2022-10-21

**Authors:** Maysam Mansouri, Preetam Guha Ray, Nik Franko, Shuai Xue, Martin Fussenegger

**Affiliations:** Department of Biosystems Science and Engineering, ETH Zurich, Basel, Switzerland; Department of Biosystems Science and Engineering, ETH Zurich, Basel, Switzerland; Department of Biosystems Science and Engineering, ETH Zurich, Basel, Switzerland; Department of Biosystems Science and Engineering, ETH Zurich, Basel, Switzerland; Department of Biosystems Science and Engineering, ETH Zurich, Basel, Switzerland; Faculty of Science, University of Basel, Mattenstrasse 26, CH-4058, Basel, Switzerland

## Abstract

The development of novel strategies to program cellular behaviors is a central goal in synthetic biology, and post-translational control mediated by engineered protein circuits is a particularly attractive approach to achieve rapid protein secretion on demand. We have developed a programmable protease-mediated post-translational switch (POSH) control platform composed of a chimeric protein unit that consists of a protein of interest fused via a transmembrane domain to a cleavable ER-retention signal, together with two cytosolic inducer-sensitive split protease components. The protease components combine in the presence of the specific inducer to generate active protease, which cleaves the ER-retention signal, releasing the transmembrane-domain-linked protein for trafficking to the trans-Golgi region. A furin site placed downstream of the protein ensures cleavage and subsequent secretion of the desired protein. We show that stimuli ranging from plant-derived, clinically compatible chemicals to remotely controllable inducers such as light and electrostimulation can program protein secretion in various POSH-engineered designer mammalian cells. As proof-of-concept, an all-in-one POSH control plasmid encoding insulin and abscisic acid-activatable split protease units was hydrodynamically transfected into the liver of type-1 diabetic mice. Induction with abscisic acid attenuated glycemic excursions in glucose-tolerance tests. Increased blood levels of insulin were maintained for 12 days.

## INTRODUCTION

Synthetic biology-inspired cell-based therapy often relies on designer cells that are able to sense a user-defined input signal, process it and respond in a customized, pre-programmed way (1,2). Many synthetic genetic circuits have been developed that allow engineered cells to produce controlled dosages of therapeutic agents on demand in response to disease-related biomarkers (3–7), chemical compounds (8–10) or physical inducers (7,11–14). However, most of these circuits are based on gene switches that control transcription of a desired therapeutic transgene from a synthetic expression unit (1). Genetic circuits with transcriptional control are useful in several contexts, but they require a considerable time to induce transcription and translation of signal-transducing proteins, and downstream signal transduction involves a further delay. Non-transcriptional responses, on the other hand, can occur within minutes and rely on post-translational controls, without the need for transcription induction or de novo protein synthesis (15). Post-transcriptional strategies offer a much higher temporal resolution than traditional transcription-based circuits, enabling direct control of the secretion of already-produced proteins in a fast and robust way (16). Moreover, protein-based circuits can be engineered to be finely tuned by small molecules or physical stimuli (17). Notably, synthetic and orthogonal proteins can be exploited to program new behaviors under desired circumstances. For example, orthogonal proteases from different species are a valuable biomedical tool since they can recognize a specific amino acid sequence in an engineered protein and cleave specific peptide bonds (18,19). Interestingly, control of protease function has already been used to engineer logic-based functions and to enable precise control of cellular processes in mammalian cells (20–22).

Inducers for on-demand control of cell behavior may be chemical or physical in nature (23). Although chemical inducers (e.g. organic and inorganic compounds, peptides, odorants) commonly provide a high level of induction and are easy to use, their potential clinical applications are often limited due to unwanted side effects, insufficient bioavailability or inappropriate pharmacodynamics (24). In contrast, traceless physical cues such as light and electric fields provide a robust, efficient and safe way to remotely control and program cellular behaviors precisely at a desired time and place (25–27).

Here, we present a programmable protease-based protein circuit to control secretion of a protein of interest after translation. This system relies on production of the desired protein in a chimeric format fused to an ER-retention signal (KKYL), which makes the protein ER-resident in the resting condition. Then, upon stimulation with an inducer, two cytoplasmic split protease components combine to generate active protease, which selectively removes the ER-retention signal, triggering trafficking and secretion of the ER-stored protein. We confirmed that this programmable protease-mediated post-translational switch (POSH) control platform can provide efficient, tunable and robust protein secretion in response to a range of chemical and physical inducers, including light and electric fields, in various mammalian and human cell types. Furthermore, as a proof-of-concept, we show that the POSH-controlled insulin release can effectively treat diabetes in a mouse model of type-1 diabetes (T1D).

## MATERIAL SAND METHODS

### Molecular cloning and DNA constructs

Details of the design and construction of the expression vectors are provided in Supplementary Tables S1 and S2.

### Cell culture and transient transfection

Human embryonic kidney cells (ATCC: CRL3216, HEK-293T), HeLa cells (ATCC: CCL-2) and CV-1 (simian)-derived cells carrying SV40 (ATCC: CRL1651, COS-7), HepG2 (ATCC: HB-8065) were cultured in Dulbecco's modified Eagle's medium (DMEM; Life Technologies, Carlsbad, CA, USA) supplemented with 10% (v/v) fetal bovine serum (FBS; Sigma-Aldrich, Munich, Germany) and 1% (v/v) penicillin/streptomycin solution (P/S: Sigma-Aldrich, Munich, Germany). All cells were cultured in a humidified atmosphere containing 5% CO_2_ at 37°C. Cell viability and number were assessed with an electric field multi-channel cell-counting device (CASY Cell Counter and Analyzer Model TT; Roche Diagnostics GmbH, Basel, Switzerland).

For *in vitro* experiments, 1.5 × 10^6^ cells in 12 ml DMEM were seeded into 96-well plates (167008, Thermo Fisher Scientific) 24 h before transfection. The transfection mix per well consisted of 200 ng of plasmid DNA mixed with 50 μl reduced-serum OptiMEM media (Gibco™, USA) and 500 ng of polyethyleneimine (PEI; Polysciences Inc.; 1 mg ml^−1^, DNA:PEI = 1:2.5). The transfection mix was prepared, vortexed, incubated for 20 min and then added to the inner 60 wells of the cell culture plate. Medium with the transfection mix was exchanged after 15 h for 125 μl/well medium with different concentrations of the appropriate inducer or no inducer as a negative control.

Transfection of _opto_POSH components was done in 24-well plates. The number of plated cells, DNA and PEI were adopted to a 24-well plate format by increasing all transfection components by a factor of 4. Cells were kept in dark immediately after transfection and protected from light thereafter. Transfection of _electro_POSH components was performed in a 6-well plate. The number of plated cells, DNA and PEI were adopted to a 6-well plate format by increasing all transfection components by a factor of 16. At 12 h after transfection, cells were trypsinized and 1.0 × 10^5^ cells were cultured in an electric cell culture chip in a similar manner to that described above.

### Chemical stimulation

Rapamycin (cat. no. 553210-10MG), abscisic acid (cat. no. A4906-1MG), gibberellic acid (cat. no. 48880–250MG) and vanillic acid (cat. no. 94770-10G) were purchased from Sigma and reconstituted in DMSO. Cells transfected with POSH control components were induced with the indicated amount of inducer or equimolar DMSO as a control.

### Light experiments

At 12 h after transfection, the cell supernatant was replaced with fresh medium containing FBS with P/S (full medium) in a light-protected condition and the cells were incubated for an additional 24 h. The medium was again replaced with fresh full medium, and the cells were irradiated with blue LEDs (475 nm, B56L5111P) as described before (28).

### Electrostimulation experiment

Cells transfected with _electro_POSH components were cultured for 24 h and electro-stimulated using a two-dimensional platform as described before (11). Prior to stimulating the cells, the culture medium was replaced with standard DMEM high glucose medium supplemented with FBS without antibiotic. The monolayer of _electro_POSH-equipped cells was stimulated by powering the cell culture chips with an amplifier-connected HP3245A Universal Source function generator. The stimulation was carried out at various values of voltage (0–50 V) and pulse length (1–100 ms peak to peak) for different time periods in order to identify the optimum conditions for the electro-induction process. After induction, electro-stimulated cells were incubated in the stimulation medium for 24 h to allow release of the protein of interest, SEAP, which was quantified as described below. After a further 24 h, the stimulation medium was replaced with medium containing resazurin sodium salt (60 $\mu$g/ml) (cat. no. R7017, Sigma-Aldrich, Saint Louis, MO, USA) and the cells were further incubated for 2 h for assessment of viability. The supernatant was analyzed (590/20 nm emission, 560/9 nm excitation) using a Tecan Infinite 200 Pro plate reader (Tecan Group AG, Maennedorf, Switzerland).

### SEAP assay

For the quantification of SEAP, the cell culture supernatant was heat-inactivated for 30 min at 65°C. Then, 20 μl of supernatant was diluted with 80 μl dH_2_O and mixed with 80 μl 2 × SEAP buffer (20 mM homoarginine, 1 mM MgCl_2_, 21% (v/v) diethanolamine, pH 9.8) and 20 μl of substrate solution containing 20 mM pNPP (Acros Organics BVBA). The absorbance at 405 nm was measured using a Tecan microplate reader (TECAN AG, Maennedorf, Switzerland). SEAP production *in vivo* was quantified with a chemiluminescence SEAP reporter gene assay (cat. no. 11779842001, Sigma-Aldrich) according to the manufacturer's instructions. Background SEAP values were not subtracted.

### Microscopy

For microscopic analysis, cells were plated in black 96-well plates, F-bottom (Greiner bio one, lot # E18053JY), treated with poly-l-lysine (Sigma P4707). Analysis was performed 6 h after stimulation. Cells were fixed with 4% paraformaldehyde in PBS and stained with SPY650-DNA (Spirochrom, USA). Imaging was performed on a Leica SP8 laser scanning confocal microscope. BFP was excited with the 405 nm laser line and emission was collected from 430 to 450 nm (405/430–450). Other fluorescent proteins were analysed under the following conditions: GFP (514/525-545), mCherry (543/585-620), SPY650 (650/640-720).

### Analytical assays

For the glucose tolerance test (GTT), mice were challenged by intraperitoneal injection of glucose (2 g/kg body weight in H_2_O) and the glycemic profiles were obtained by measurement of blood glucose levels with a glucometer (Contour® Next; Bayer HealthCare, Leverkusen, Germany) every 15 or 30 min for 120 min. The viability of POSH-transfected cells following electrostimulation was checked by resazurin assay (cat. no. R7017, Sigma-Aldrich, Saint Louis, MO, USA). Mouse insulin levels (mINS) in cell culture medium and mouse serum were quantified with a Mouse Insulin ELISA kit (Mercodia; cat. no. 10–1247-01) and an Ultrasensitive Mouse Insulin ELISA kit (Mercodia; cat. no. 10-1249-01), respectively.

### Western blotting

HEK-293T cells were transfected in 6-well plates and grown overnight. Cells were treated without (DMSO) or with 100 nM rapamycin in complete medium, collected after 0, 12 and 24 h, and lysed with RIPA buffer (50 mM Tris pH 7.6, 150 mM NaCl, 1% Triton X-100, 1% Na-deoxycholate, 0.1% SDS) containing freshly added complete protease inhibitor cocktail (Roche) and 0.5 μl Benzonase® endonuclease (Novagen). After centrifugation for 10 min at 18 000 × g and 4°C, the protein concentration of the supernatant was quantified using a BCA assay (ThermoFisher) and an aliquot containing 10 μg protein was run on a Bolt 4–12% Bis-Tris gel (ThermoFisher). Proteins were transferred to nitrocellulose membranes (Amersham) according to standard procedures. Mouse anti-HA (Sigma, H3663; diluted 1 : 1000 in 5% non-fat dried milk/PBST) and rabbit anti-FLAG (Abcam, ab205606; diluted 1:1000 in 5% non-fat dried milk/PBST) were used as primary antibodies. As secondary antibodies, alkaline phosphatase-coupled ECLTM anti-mouse and anti-rabbit IgGs (GE Healthcare, diluted 1:10 000 in 5% non-fat dried milk/PBST) were used, followed by chemiluminescence detection. Images were acquired using a Fusion FX apparatus (Vilbert Lourmat). Quantification was performed with ImageJ.

### Cell encapsulation

Transiently co-transfected engineered HEK293T cells were encapsulated in 400 mm alginate-PLL (poly-L-lysine)-alginate beads using the Inotech Encapsulator Research IE-50R (Buechi Labortechnik AG, Flawil, Switzerland) according to the manufacturer's protocol. The parameters were as follows: 25 ml syringe operated at a flow rate of 500 units, 200 mm nozzle with a vibration frequency of 1024 Hz and bead dispersion voltage of 1.2 kV, stirrer speed set at 4.5 units.

### Animal study

All experiments involving animals were performed in accordance with the Swiss animal welfare legislation and approved by the Veterinary Office of the Canton Basel-Stadt (approval no. 2879/31996).

### Experimental animals

Male wild-type C57BL/6JRj mice, aged 8–9 weeks were obtained from Janvier Labs (Saint-Berthevin, France) and acclimatized for at least 1 week. Type-1 diabetic model mice were generated by intraperitoneal injection of STZ for 5 days at a dose of 60 mg kg^−1^ per day in male C57BL/6J mice weighing at least 25 g. One week after STZ injection, fasting blood glucose and fasting insulin levels were monitored in STZ-treated mice and compared with those of wild-type mice. STZ-treated mice with a level of blood glucose higher than 15 mM and a fasting insulin level lower than that of wild-type mice were considered as diabetic. Animals were housed with an inverse 12 h day-night cycle in a temperature (21 ± 2°C) and humidity (55 ± 10%)-controlled room with ad libitum access to standard diet and drinking water. Animals were randomly assigned to experimental groups.

### Implantation experiments

Implantation in mice was performed by subcutaneous injection of 1 ml glucose-free DMEM containing alginate-poly(L-lysine)-alginate-microencapsulated POSH-equipped engineered cells at the side of the back. Plasma was collected by centrifugation (10 min, 5000 g) of clotted blood (20 min at 37°C and then 20 min at 4°C).

### Hydrodynamic injection of plasmid DNA into mice

Mice were placed under a heating lamp for 10 min, then sterile saline solution containing a total of 100 μg DNA was injected through the tail vein within 10 s (29).

### Abscisic acid (ABA) administration

Abscisic acid (TOCRIS, cat. no. 6554) was dissolved in dimethyl sulfoxide (DMSO), and this solution was mixed with sterile saline solution (0.9% w/v sodium chloride) at a ratio of 1:5 (v/v). A total of 300 μl of the resulting ABA formulation was interperitoneally injected into each mouse. Negative control mice in this study received the same treatment, except that DMSO alone was used in place of the ABA solution. Blood was collected for SEAP measurement at 6 h and 12 h after the injection.

### Statistical analysis

Statistical tests and significance are reported in the figures and corresponding figure legends. A two-tailed, paired Student's *t*-test and one-way or two-way ANOVA with Tukey's test were applied to determine the statistical significance of differences among groups, using GraphPad Prism. *P < 0.05; **P < 0.01; ***P < 0.001; ****P < 0.0001.

## RESULTS

### Design of the POSH control platform

**Figure 1.**
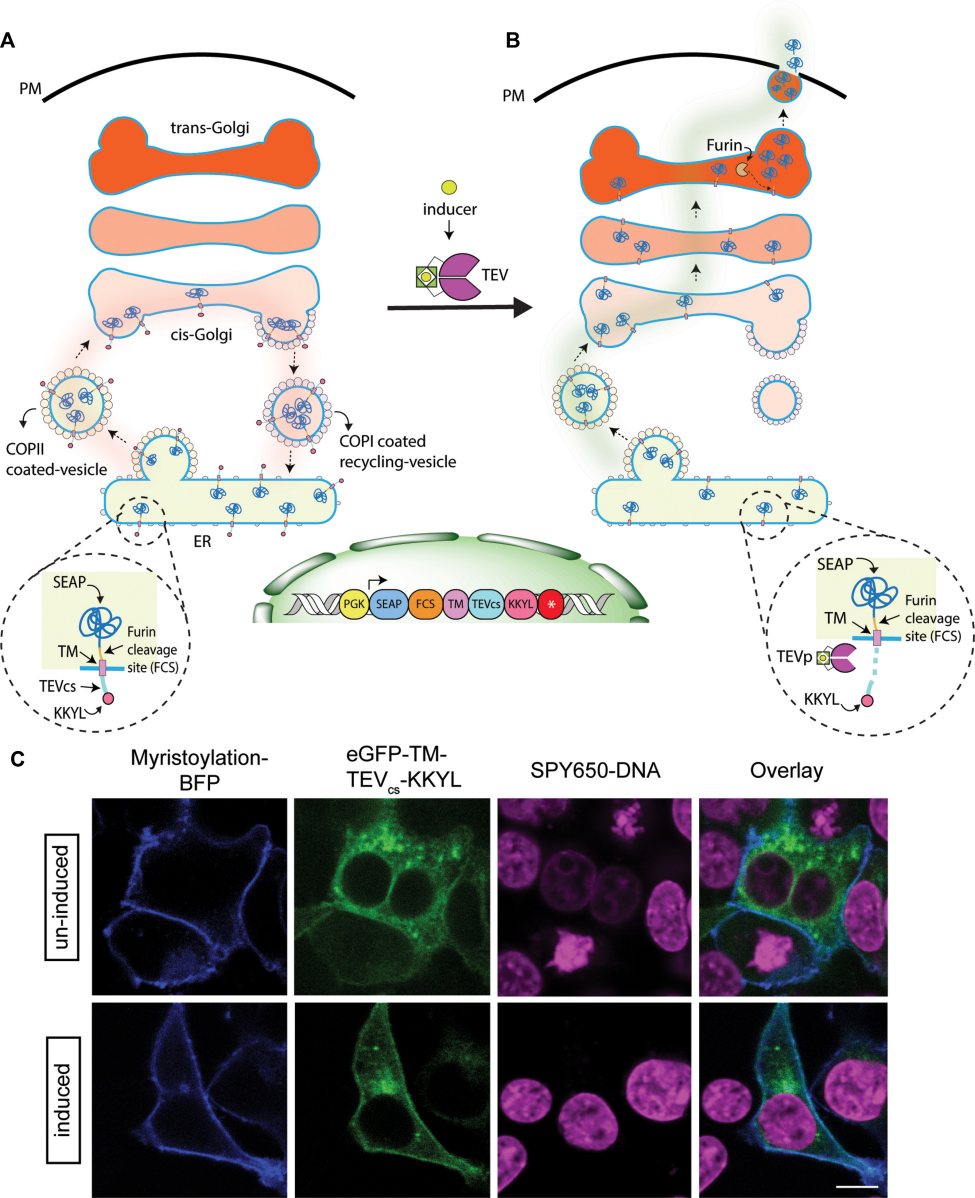
Design of the programmable protease-mediated post-translational switch (POSH) platform. (**A**) Schematic representation of the POSH control concept and its elements. The POSH control platform is composed of a split protease-based inducible controller unit as well as a constitutively expressed synthetic reporter unit. In the unstimulated state, the engineered reporter unit expresses a protein of interest fused via a transmembrane domain to an ER-retention signal, which is located in the cytoplasm. The ER retrieval signal binds to COPI-coated vesicles, ensuring their return from cis-Golgi to the ER through a recycling pathway. (**B**) A user-defined stimulus (chemicals, light or electrostimulation) triggers activation of the split protease-controller unit, leading to removal of the ER retrieval signal from the synthetic reporter, which can then be trafficked to the trans-Golgi region, where endogenous furin cleaves the desired protein for secretion the outside of the cell. (**C**) Microscopic images of HEK293T cells transfected with pMMZ686 (P_SV40_-Myristoylation_SS_-BFP-pA_P_PGK_-Igk-eGFP-TM-TEVcs_(3x)_-KKYL-STOP_-_pA_P_hCMV_-FLAG-NES-FRB-nTEVp-pA_P_hCMV_-FLAG-NES-FKBP-cTEVp-pA) and fixed before and after 6 h stimulation with 1 μM rapamycin. Cells were stained with SPY650-DNA. Myristoylation_SS_-BFP tagged the plasma membrane and was used to demonstrate colocalization of eGFP and the plasma membrane in unstimulated and stimulated states. Scale bar is 10 μm. A representative image of three replicates from each group is shown. Source data are provided as a Source Data file.

The POSH control system contains three components; a synthetic reporter unit carrying a protein of interest, together with two split protease controller units which combine to form a functional protease upon stimulation with the specified inducer. The synthetic reporter unit is under the control of a constitutively active phosphoglycerate kinase (PGK) promoter that drives expression of a chimeric construct consisting of a secretable protein of interest linked to a protease cleavage site (e.g. TEVcs) followed by an ER-retention signal (KKYL). We positioned the protein of interest inside the ER and the protease-cleavage site outside the ER by inserting a plasma transmembrane (TM) domain between the two. Also, a furin site (RNRQKR) was introduced downstream of the protein of interest to ensure cleavage and subsequent release of the desired protein after translocation to the trans-Golgi region (Figure 1). Mammalian cells transfected with a synthetic reporter express the desired protein in the ER lumen while the protease cleavage site and retention signal face the cytoplasm. The ER retrieval signal binds to COPI-coated vesicles, facilitating return to the ER through a recycling pathway in the resting state (Figure 1A). In the presence of a user-defined stimulus (e.g. chemicals, light or electric fields), the engineered split-protease-based controller units bind together to form a functional enzyme that recognizes the cleavage site and removes the ER-retention signal. This allows trafficking of remaining protein to the trans-Golgi region, where it is further processed by endogenous furin and then secreted to the outside of the cell (Figure 1B).

To study translocation of the stored protein from ER to trans-Golgi and plasma membrane, we used eGFP as a synthetic reporter (without the furin site). eGFP was mainly accumulated in the ER in the unstimulated condition, while it was mostly exposed on the plasma membrane after stimulation (Figure 1c and Supplementary Figures S1 and S2).

### POSH control platform

**Figure 2.**
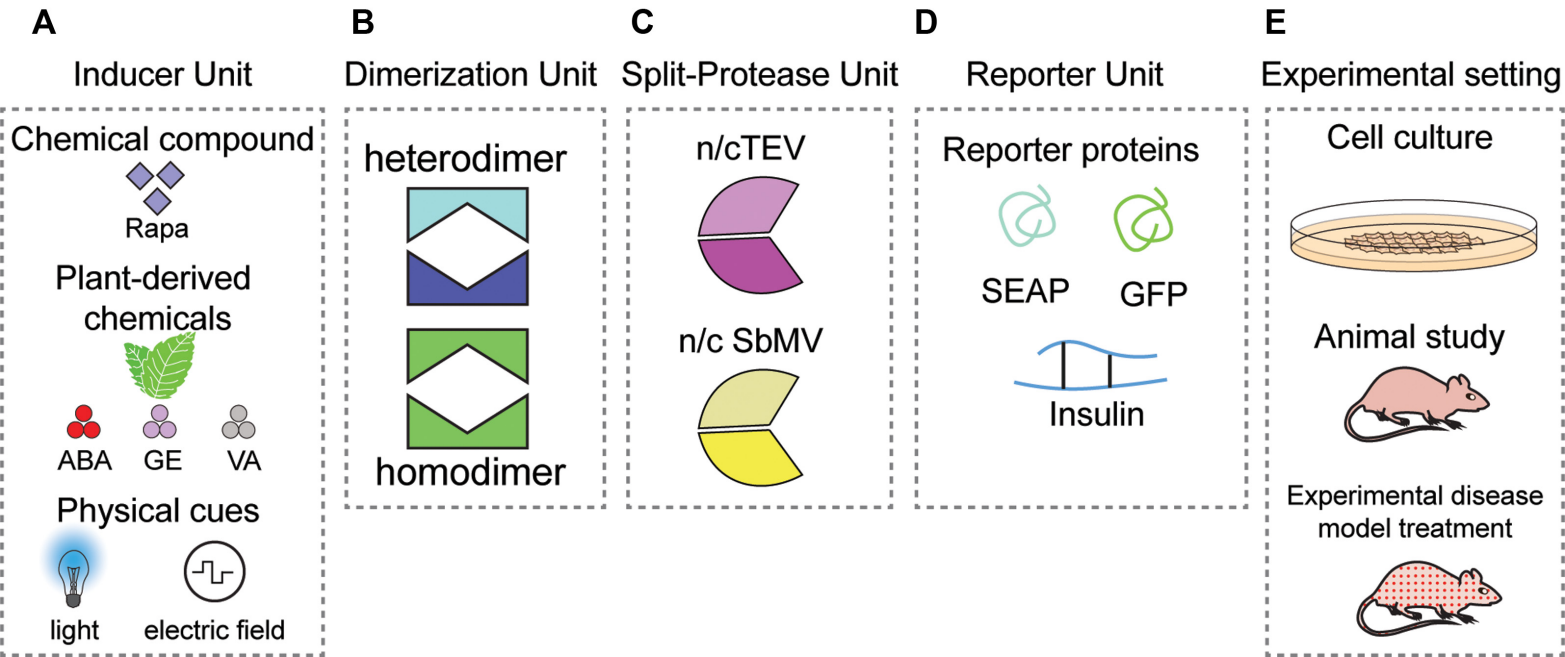
POSH control platform. (**A**) Various user-designed inputs, including rapamycin, plant-derived small molecules, and traceless inducers such as light and electric fields, can be used to program the POSH control system in mammalian cells. (**B**) The inducer triggers interaction between binding domains in the controller units, which can be hetero- or homo-dimers. (**C**) Proteases from various species can be activated to specifically cut the desired site on the synthetic reporter upon stimulation. (**D**) The synthetic reporter unit expresses the selected protein, such as SEAP or eGFP as a reporter protein or insulin as a therapeutic protein, which is subsequently secreted. (**E**) POSH control can be implemented in cell culture or animal experiments to provide on-demand secretion of the protein of interest.

To build a post-translational switch control platform, we engineered POSH control components that would enable designer cells to sense a variety of stimuli, including rapamycin, plant-derived chemicals, light and electrostimulation (Figure 2A). To achieve this, different homo- and hetero-dimerization domains (Figure 2B) are linked to various split proteases (Figure 2C), enabling cleavage and subsequent secretion of the stored protein (Figure 2D) either in cell culture or in animals (Figure 2E).

### Characterization of POSH control

**Figure 3.**
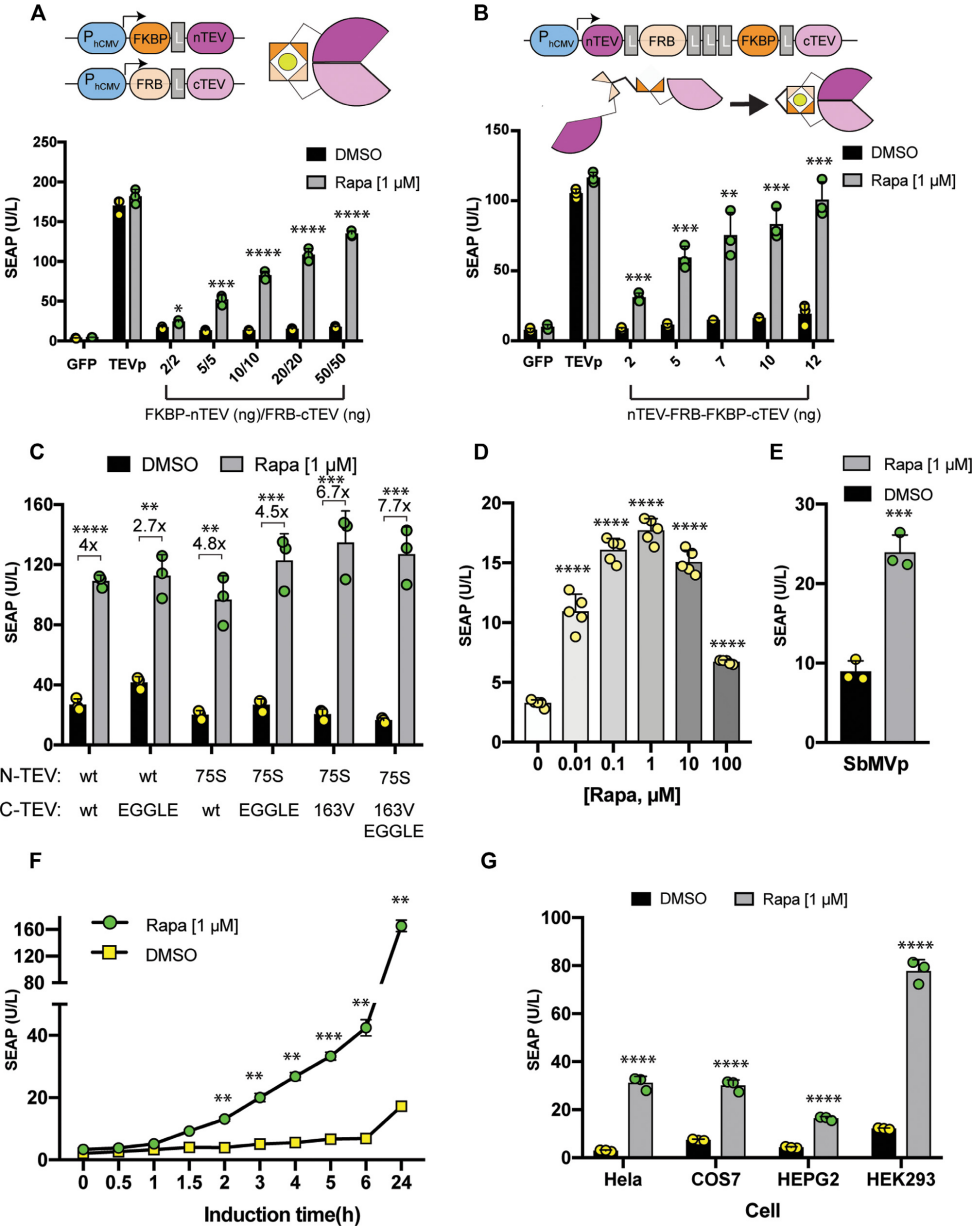
Characterization of POSH-transfected cells. HEK293T cells were transiently co-transfected with pMMZ1363 (P_PGK_-SEAP-Furin_(3x)_-TM-TEVcs_(3x)_-KKYL-STOP_-_pA), and the indicated rapamycin-responsive plasmids. Cells were treated with either 1 μM rapamycin (induced group) or equimolar DMSO (control group). SEAP levels were assessed in the supernatant of cultured cells after 24 h, unless otherwise indicated. (**A**) Co-transfection using individual plasmids pMMZ687 (P_hCMV_-FKBP-G_4_S-nTEVp_-_pA) and pMMZ688 (P_hCMV_-FRB-G_4_S-cTEVp_-_pA) encoding the protease-based controller unit. (**B**) Co-transfection of ‘all-in-one’ plasmid pMMZ507 (P_hCMV_-nTEVp-G_4_S-FRB-(G_4_S)_3x_-FKBP-G_4_S-cTEVp_-_pA). (**C**) Efficiency of mutated n/cTEVp versus the wild type (wt) to cleave and release SEAP. (**D**) Dose-dependent activity of the POSH control platform in the presence of increasing concentrations of rapamycin. Cells were transiently co-transfected with 50 ng of each pMMZ693 (P_hCMV_- FLAG_3x_-NES-FRB-G_4_S-cTEVp _(EGGLE/163v)_-pA) and pMMZ694(P_hCMV_- FLAG_3x_-NES-FRB-G_4_S-nTEVp_(75S)_-pA) along with 100 ng of pMMZ1363. (**E**) SbMV protease version of POSH control. HEK293T cells were co-transfected with pMMZ565 (P_PGK_-SEAP-Furin_(3x)_-TM-SbMVcs_(3x)_-KKYL-STOP_-_pA), P_hCMV_-FRB-nSbMV_-_pA and P_hCMV_-FKBP-cSbMV_-_pA constructs and SEAP secretion was induced by rapamycin. (**F**) *In vitro* secretion kinetics. POSH-transfected HEK293T cells were induced by rapamycin for various periods as indicated and the level of secreted SEAP was measured in the supernatant of cultured cells. (**G**) Ability of different POSH-equipped mammalian cell types to release SEAP upon induction by rapamycin. Bars show the mean ± s.d. of *n* = 3 biologically independent samples, with the individual data points. Statistical significance of differences between stimulated and un-stimulated groups was calculated using an unpaired Student's *t*-test. Numbers above the bars indicate fold changes of reporter (SEAP) expression versus the corresponding control group. *P< 0.05; **P< 0.01; ***P< 0.001; ****P< 0.0001. Source data are provided as a Source Data file. L; linker (GGGGS).

To characterize the POSH control system, we capitalized on the well-known rapamycin-activated FKBP/FRB heterodimerization system (30) fused to split TEVp (FKBP-nTEVp/FRB-cTEVp). Expression of POSH control components in HEK cells resulted in significant induction of the reporter, SEAP (human placental secreted alkaline phosphatase), in the presence of rapamycin (Figure 3A and Supplementary Figure S3). To facilitate delivery of the inducible protease units, we designed an ‘all-in-one’ construct in a tandem format; this provided similar inducibility with a lower required total amount of DNA (Figure 3B). We also introduced different mutations in the split n/cTEVp to increase the specificity of cleavage and to increase the total induction fold by decreasing leakiness (Figure 3C) (31–33). The level of protein secretion could be fine-tuned by applying different concentrations of rapamycin (Figure 3D). We confirmed that treatment with rapamycin did not influence n/cTEVp expression at the protein level (Supplementary Figure S4). Exchanging split n/c TEVp with n/cSbMVp allowed us to retarget the POSH control system to a synthetic reporter containing an SbMVp cleavage site, thereby inducing SEAP secretion upon rapamycin administration (Figure 3E). We also studied the kinetics of protein secretion and demonstrated that a significant level of protein release can be achieved within 2–3 h of stimulation (Figure 3F). Therefore, protein release under POSH Control is slower than that from fast-releasing systems (e.g. depolarization-based approaches,(11) which show peak secretion at 10–15 min after the start of induction), but faster than that from transcriptional-based control systems (8–12 h after the start of induction) (Supplementary Figure S5). The POSH control system also showed excellent inducibility when it was engineered into various other types of mammalian cells (Figure 3G).

### Plant-derived chemical-activated POSH control

**Figure 4.**
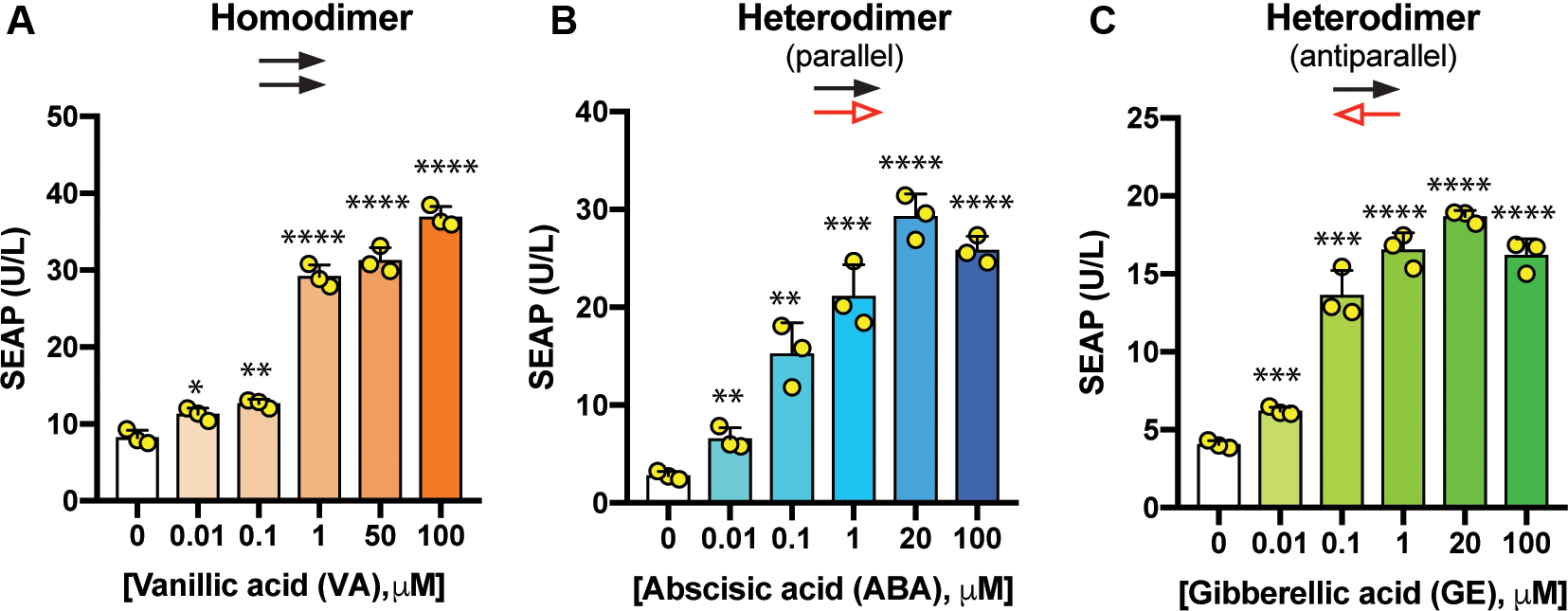
Programming POSH control with plant-derived chemicals. Activation of the POSH control system with different plant-derived chemicals. (**A**) HEK293T cells transfected with pMMZ1363 (P_PGK_-SEAP-Furin_(3x)_-TM-TEVcs_(3x)_-KKYL-STOP_-_pA), pMMZ641 (P_hCMV_-VanR-G_4_S-nTEVp-pA), and pMMZ639 (P_hCMV_-VanR-G_4_S-cTEVp-pA) were treated with vanillic acid. (**B**) HEK293T cells transiently co-transfected with POSH control constructs (pMMZ1363, pMMZ574; P_hCMV_-ABI-G_4_S-cTEVp-pA, and pMMZ575; P_hCMV_-PYL1-G_4_S-nTEVp-pA) were treated with abscisic acid. (**C**) HEK293T cells co-transfected with pMMZ1363, pMMZ637; P_hCMV_-GAI-G_4_S-cTEVp-pA, and pMMZ666; P_hCMV_-nTEVp-G_4_S-GID-pA were treated with gibberellic acid. Transfected cells were stimulated with the indicated concentrations of inducers and SEAP levels in the supernatant of cultured cells were profiled at 24 h. Bars show the mean ± s.d., of *n* = 3 biologically independent samples, with the individual data points. The significance of differences between stimulated and un-stimulated groups was calculated using an unpaired Student's *t*-test. Source data are provided as a Source Data file.

In order to control POSH with the chemicals known to be safe for usage in human, we focused on three plant-derived clinically compatible small molecules. We fused either homodimeric vanillic acid (VA) (34) or heterodimeric abscisic acid (ABA) (35) and gibberellic acid (GE) (36) interacting domains in different orientations to the n/cTEVp (VanR-nTEVp/VanR-cTEVp, PYL1-nTEVp/ABI-cTEVp and nTEVp-GID/GAI-cTEVp) and observed secretion of SEAP protein upon administration of the relevant chemical in a dose-dependent manner (Figure 4A–C and Supplementary Figure S6). Heterodimerizing systems (ABA and GE) showed a higher level of induction than a homodimerizing domain (VA).

### Remote regulation of POSH control

**Figure 5.**
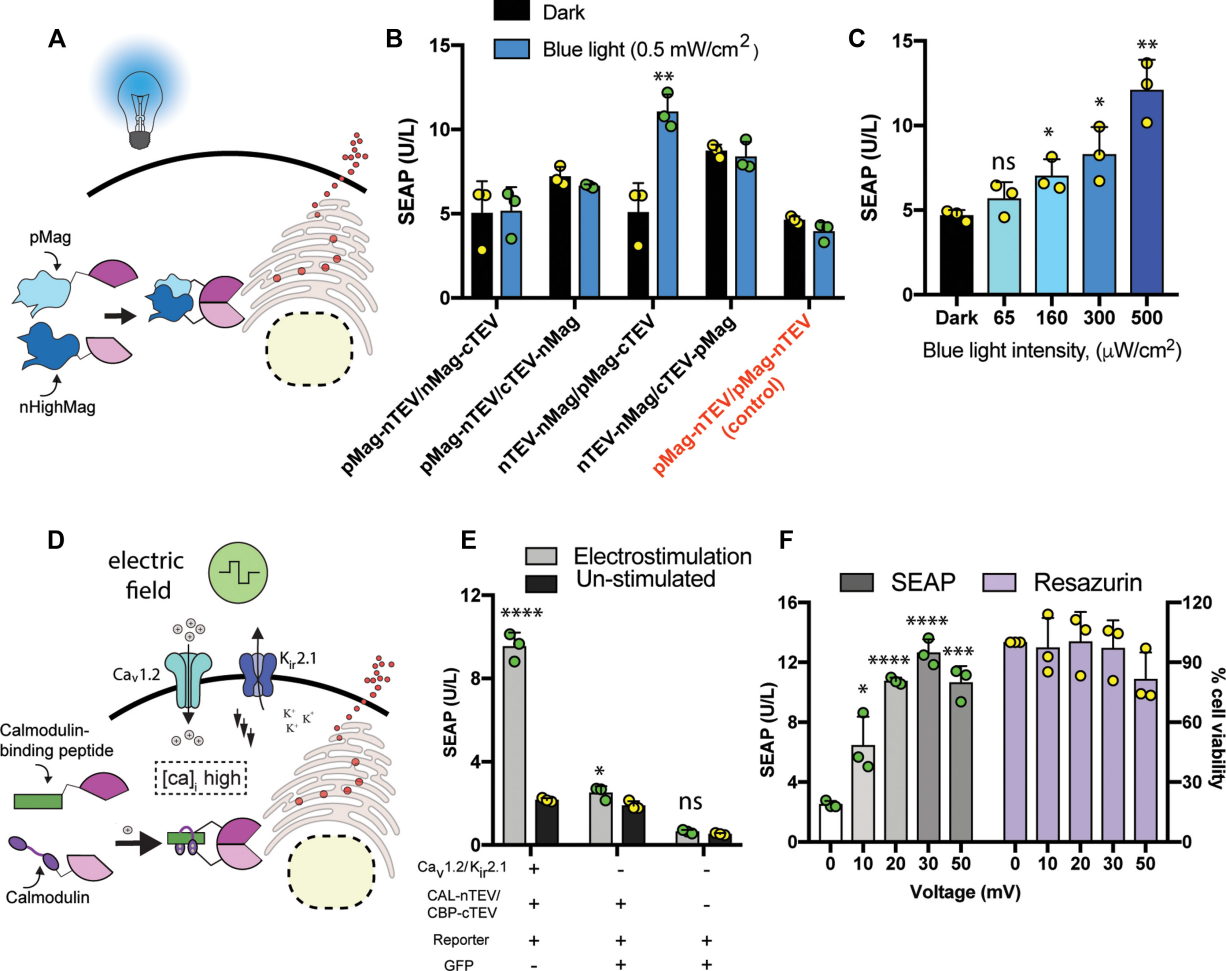
Remotely regulatable POSH control. (**A**) Schematic representation of light-inducible POSH control. For _opto_POSH, cells were transfected with a synthetic reporter unit (pMMZ1363; P_PGK_-SEAP-Furin_(3x)_-TM-TEVcs_(3x)_-KKYL-STOP_-_pA) as well as programmable split protease units fused to blue-light-sensitive pMag and nHighMag. Activated protease produced by blue light (470 nm) exposure cleaves the ER-retention signal and eventually enables transportation and secretion of SEAP. (**B**) Various orientations of the protease domains and blue-light-responsive elements were tested. (**C**) HEK293T cells transfected with pMMZ1363, pMMZ678; P_hCMV_-nTEVp-G_4_S-nHighMag-pA, and pMMZ662; P_hCMV_-pMag-G_4_S-cTEVp-pA. Cells were illuminated with blue light of different intensities, and the secreted SEAP levels in the supernatant of cultured cells were profiled at 24 h. (**D**) Schematic representation of electrostimulation-triggered POSH control. For _electro_POSH, cells were transfected with voltage-gated channels (pKK66; P_hEF1α_-α_1_C-P2A-K_ir_2.1-pA and pKK56; P_hEF1α_-α_2_/δ_1_-P2A-β_3_-pA) as well as calcium-sensitive controller units (pMMZ560; P_hCMV_-calmodulin-G_4_S-nTEVp-pA and pMMZ561; P_hCMV_-calmodulin binding peptide (CAMB2M)-G_4_S-cTEVp-pA) along with synthetic reporter pMMZ1363. An electric field triggers opening of Ca_v_1.2, which initiates calcium influx into the cytoplasm. This leads to interaction of the calcium-responsive units and secretion of the desired protein. (**E**) Dependence of activation of _electro_POSH on the presence of each element. (**F**) SEAP secretion and cell viability upon stimulation with the indicated voltages. Bars show the mean ± s.d. of *n* = 3 biologically independent samples, with the individual data points. The significance of differences between stimulated and un-stimulated groups was calculated using an unpaired Student's t-test. Source data are provided as a Source Data file.

To establish a traceless strategy for regulating the POSH control platform wirelessly, we focused on light and electric fields, and developed _opto_POSH control and _electro_POSH control modules, respectively. A light-inducible approach was developed by engineering blue-light-responsive domains of pMag/nHighMag (37) to n/cTEVp (Figure 5A). We showed an antiparallel orientation produces functional TEVp upon blue light illumination, and induces SEAP secretion (Figure 5B). Light-triggered secretion of SEAP under the _opto_POSH control system can also be fine-tuned by modulating the light intensity (Figure 5C). The _electro_POSH control system was obtained through co-expression of voltage-gated ion channel Ca_V_1.2 and inwardly rectifying potassium ion channel K_ir_2.1 (11) along with calcium-responsive POSH components including calmodulin and calmodulin-binding peptide (38) fused to TEVp (calmodulin-nTEVp/calmodulin-binding peptide-cTEVp) and the synthetic reporter carrying SEAP. Voltage-gated ion channel circuits can sense electric fields and open Ca_V_1.2, enabling calcium influx to the cell cytoplasm. The high level of intracellular calcium triggers interaction of calcium-responsive POSH control elements to form functional TEVp, which cleaves the ER-retention signal and induces protein secretion (Figure 5D). We showed that _electro_POSH-mediated secretion of SEAP occurs only when all necessary components are expressed (Figure 5E). Furthermore, the level of protein secretion can be regulated by varying the applied voltage (Figure 5F), the electrical pulse length and the stimulation time (Supplementary Figure S4). Resazurin assay established that the applied electrical field has no adverse impact on cell viability (Figure 5F and Supplementary Figure S7).

### *In vivo* application of POSH control

**Figure 6.**
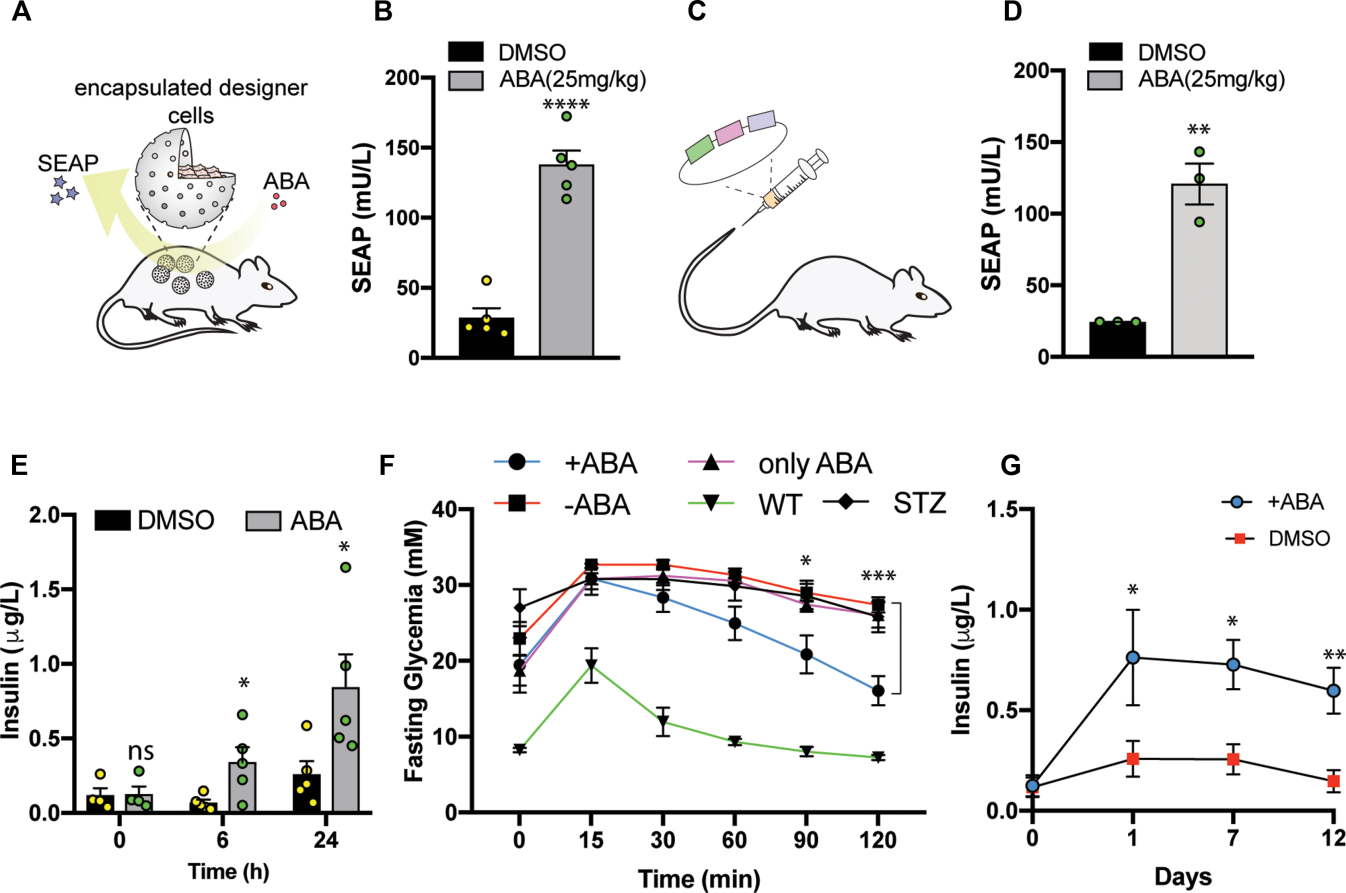
*In vivo* validation of POSH control for the treatment of experimental type-1 diabetes. (**A–D**) *In vivo* application of POSH control. a, POSH control-based cell therapy. Schematic representation of mice implanted with 10^7^ microencapsulated HEK293T cells (transiently co-transfected with pMMZ1363/pMMZ574/pMMZ575) and treated with abscisic acid (ABA, 25 mg/kg) to induce release of SEAP. b, SEAP levels in the blood were measured at 24 h. Experimental groups are indicated above the plot. Bars show mean ± SEM (n = 5), with the individual data points. c, POSH control-based gene therapy. Mice were hydro-injected with pMMZ690 (P_PGK_-SEAP-Furin_(3x)_-TM-TEVcs_(3x)_-KKYL-STOP_-_pA_P_hCMV_-ABI-G_4_S-cTEVp-pA_pMMZ575; P_hCMV_-PYL1-G_4_S-nTEVp-pA) through a tail vein. d, SEAP levels in blood of the mice were profiled at 24 h after induction with abscisic acid (ABA, 25 mg/kg). Bars show mean ± SEM (n = 3), with the individual data points. (**E–G**), POSH-controlled treatment of type-1 diabetic mice. e, POSH control-mediated insulin release *in vivo*. STZ-induced type-1 diabetic mice were hydro-injected with pMMZ689 (P_PGK_-mINS-Furin-TM-TEVcs_(3x)_-KKYL-STOP_-_pA_P_hCMV_-ABI-G_4_S-cTEVp-pA_pMMZ575; P_hCMV_-PYL1-G_4_S-nTEVp-pA) and insulin release was induced by administration of abscisic acid (ABA, 25 mg/kg). Blood levels of insulin were measured in starved diabetic mice at the indicated time points. Bars show mean ± SEM (*n* = 5), with the individual data points. (**F**), Intraperitoneal glucose tolerance test (IPGTT) was performed by administration of 2 g/kg aqueous D-glucose and was conducted 24 h after induction with abscisic acid (ABA, 25 mg/kg) in overnight-starved animals. Wild-type (WT) C57BL/6JRj mice (*n* = 5) as well as STZ-induced type-1 diabetic mice (*n* = 5) without hydro-injection of the pMMZ689 plasmid and treated with or without abscisic acid (ABA, 25 mg/kg) were used as controls. Values are mean ± SEM. The statistical significance of differences between STZ-induced type-1 diabetic groups stimulated or non-stimulated with abscisic acid (ABA, 25 mg/kg) were calculated using two-way ANOVA. (**G**) Insulin levels over a period of 12 days in the blood of STZ-induced T1D mice hydro-injected with POSH control plasmid (pMMZ689) and treated with or without abscisic acid (ABA, 25 mg/kg). Values are mean ± SEM (*n* = 5). The statistical significance of differences between indicated groups was calculated using a two-tailed, unpaired Student's t-test. ns, not significant; *P< 0.05; **P< 0.01; ***P< 0.001; ****P< 0.0001. Source data are provided as a Source Data file.

To validate POSH control performance *in vivo*, we intraperitoneally implanted microencapsulated human cells engineered for POSH-controlled, abscisic acid-induced SEAP secretion in mice. Administration of abscisic acid (Figure 6A) resulted in a significantly higher level of secreted SEAP in the bloodstream compared to control animals (Figure 6B). We also hydro-injected the all-in-one POSH control plasmid into mice and confirmed that expression of the POSH control system in the host is effective; SEAP was secreted in the presence of abscisic acid (Figure 6C, D and Supplementary Figure S8a, b).

Next, to validate our post-translational protein-based circuit in a clinical proof-of-concept study, we assembled a POSH control plasmid encoding insulin as well as abscisic acid-activated protease units in a single plasmid (Supplementary Figure S8c), which was hydrodynamically transfected into the liver of type-1 diabetic (T1D) mice. The T1D mice showed a significantly higher level of insulin at 6 h after administration of abscisic acid compared to control animals (Figure 6E). Abscisic acid induction also attenuated glycemic excursions in glucose-tolerance tests (Figure 6F) and the mice showed higher levels of insulin in their blood stream for 12 days, compared with control animals (Figure 6G).

## DISCUSSION

The POSH control platform is a protein-level control module that can directly regulate protein secretion on demand. Compared to transcription-based gene circuits, which are too slow for some therapeutic applications, POSH control features a faster response, as well as great versatility, high effectiveness and clear inducer dose-dependency. The platform can be engineered to respond to a range of chemical and physical inducers. We designed the controller unit of the POSH system to be located in the cytoplasm rather than in the ER, so that only the protein of interest (and not the control units themselves (39)) is secreted to the outside of the cells. This also avoids the need for tedious steps of mutation and adaptation of the controller units to the environment of the ER (32). Furthermore, POSH can link the protein control module to endogenous signaling events, such as calcium influx, that occur in the cytoplasm. To ensure efficient delivery of POSH, we demonstrated compact co-delivery of the necessary components in a single plasmid as well as delivery as an all-in-one chimeric form. In contrast to a recently developed strategy that provides control in the translational step by incorporating non-canonical amino acids in the protein sequence (40), our post-translational control system works in the natural cell environment without the need for any exogenous supplement, other than the inducer. Although post-translational control strategies have recently been developed that allow timely control of protein secretion (20–22,41), they are limited to chemical inducers and have so far been employed only in vitro. In contrast, we showed here that POSH control can provide on-demand secretion of insulin to normalize hyperglycemia in T1D mice. For this *in vivo* study, we used abscisic acid, a plant-derived phytohormone which has been proven to stimulate the activity of insulin-releasing pancreatic β-cells (42,43), as an inducer to trigger insulin secretion.

In addition, we showed that the POSH control system can employ traceless inducers such as light and electric fields. Such inducers can non-invasively connect optogenetic and electrogenetic interventions to real-world wearable electronic devices, opening up the possibility of real-time remote programming of cell-based therapies for disease management or lifestyle improvement (44,45). We have reported fast release designer cells that were able to secrete therapeutics in response to light (46) and electrostimulation (11). However, the engineered genetic circuits in the light and electro-triggered fast release systems were implemented in a cancer-derived β cell line (1.1E7) (47). Furthermore, those systems suffered from a high background in the resting condition, because regulation of protein release was linked to depolarization of the plasma membrane, which can be very non-specific. In contrast, the POSH control circuit can be used not only in β cell lines, but also in other mammalian cell types (Figure 3g). Hence, POSH control paves the way to expand synthetic biology-derived strategies to clinically validated human cell types, e.g. patient-derived autologous somatic cells, which should eventually increase the level of safety for translational applications (48,49). Also, the POSH platform relies on selective cleavage mediated by an orthogonal protease (20) upon stimulation, resulting in a tightly controlled system with minimum leakiness.

Collectively, these results suggest that the protein circuit-based POSH platform represents a valuable tool to control and program cell behavior at the post-translational level. We believe this compact and modular design has the potential to support robust synthetic biology-mediated therapies for diseases that requires rapid delivery of the therapeutics at crucial intervention points.

## DATA AVAILABILITY

The authors declare that all data supporting the findings of this study are available within the paper and its supplementary information files. All vector information is provided in Supplementary Table S1. Source data are provided with this paper. Requests for materials should be made to the corresponding author. All plasmids generated in this study are available upon request.

## Supplementary Material

gkac916_Supplemental_Files
