# Supplementary material for: Design of programmable post-translational switch control platform for on-demand protein secretion in mammalian cells
Source: Nucleic Acids Res. 2022 Oct 21;51(1):e1. doi: 10.1093/nar/gkac916 (PMC9841418; doi:10.1093/nar/gkac916)
Supplement: gkac916_Supplemental_Files [file gkac916_supplemental_files.zip › SI_NAR-01273-Met-G-2022.docx]

Supplementary Information

**Design of Programmable Post-Translational Switch Control Platform for On-demand Protein Secretion in Mammalian Cells**

Maysam Mansouri^1^, Preetam Guha Ray^1^, Nik Franko^1^, Shuai Xue^1^, and Martin Fussenegger^1,2*^

^1^Department of Biosystems Science and Engineering, ETH Zurich, Basel, Switzerland.

^2^Faculty of Science, University of Basel, Mattenstrasse 26, CH-4058, Basel, Switzerland. *Correspondence to [fussenegger@bsse.ethz.ch](mailto:fussenegger@bsse.ethz.ch)

^1^ Department of Biosystems Science and Engineering, ETH Zurich, Basel, Switzerland.

^2^ Faculty of Science, University of Basel, Mattenstrasse 26, CH-4058, Basel, Switzerland.

*Corresponding author. E-mail: fussenegger@bsse.ethz.ch

**
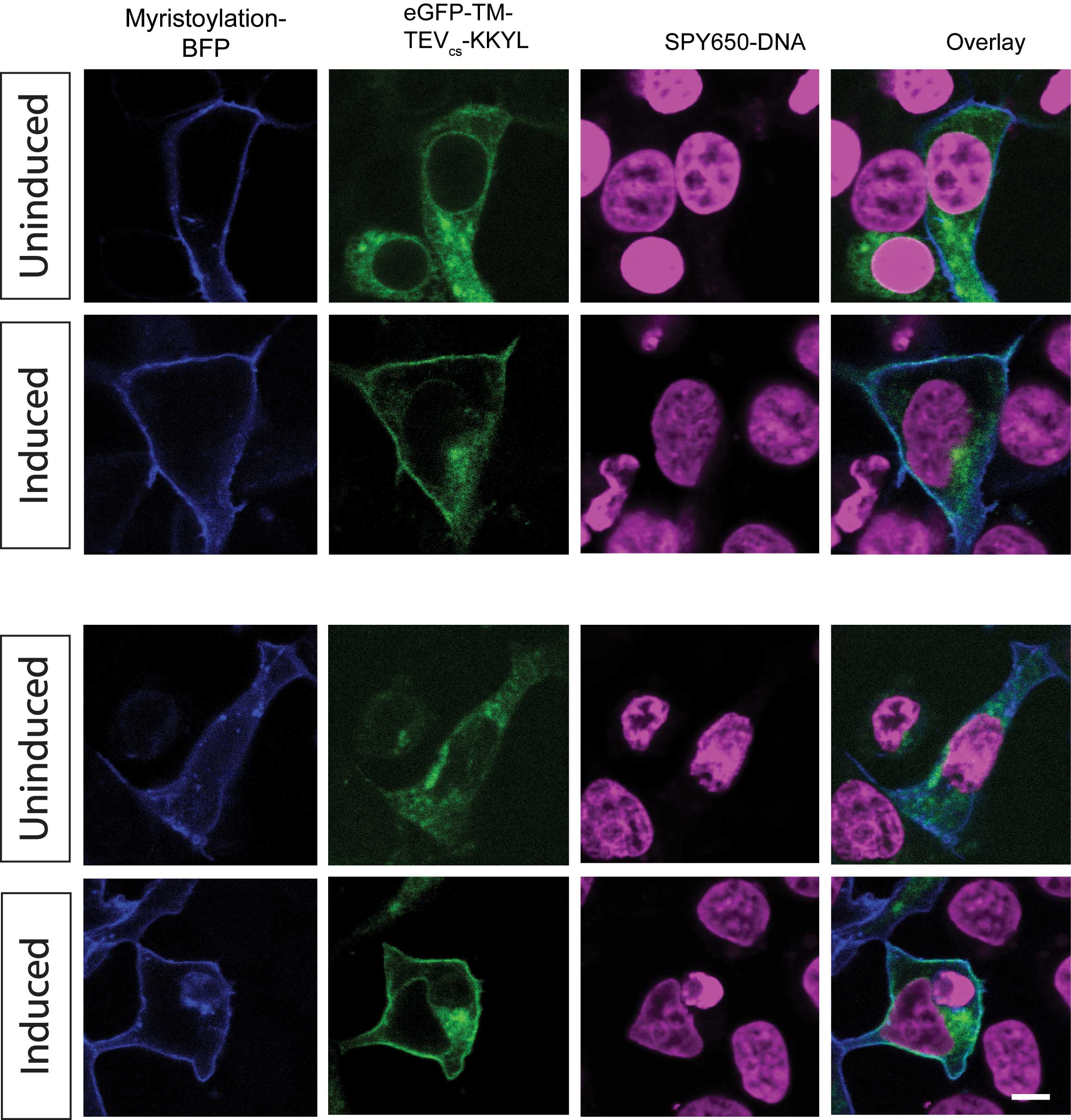
**

**Supplementary Figure 1: Microscopy analysis of the expression of POSH control components.** HEK293T cells were transfected with pMMZ686 (P_SV40_-Myristoylation_SS_-BFP-pA_P_PGK_-Igk-eGFP-TM-TEVcs_(3x)_-KKYL-STOP_-_pA_P_hCMV_-FLAG-NES-FRB-nTEVp-pA_P_hCMV_-FLAG-NES-FKBP-cTEVp-pA) and were fixed before (uninduced) and after (induced) 6 h stimulation with 1 μM rapamycin. Myristoylation _Signal sequence(ss)_-BFP (blue channel) tagged the plasma membrane and was used to demonstrate colocalization of eGFP (desired protein, green channel) and the plasma membrane in unstimulated and stimulated states Cells were stained with SPY650-DNA (purple channel, and indicates nucleus). Scale bar, 10 μm.

**
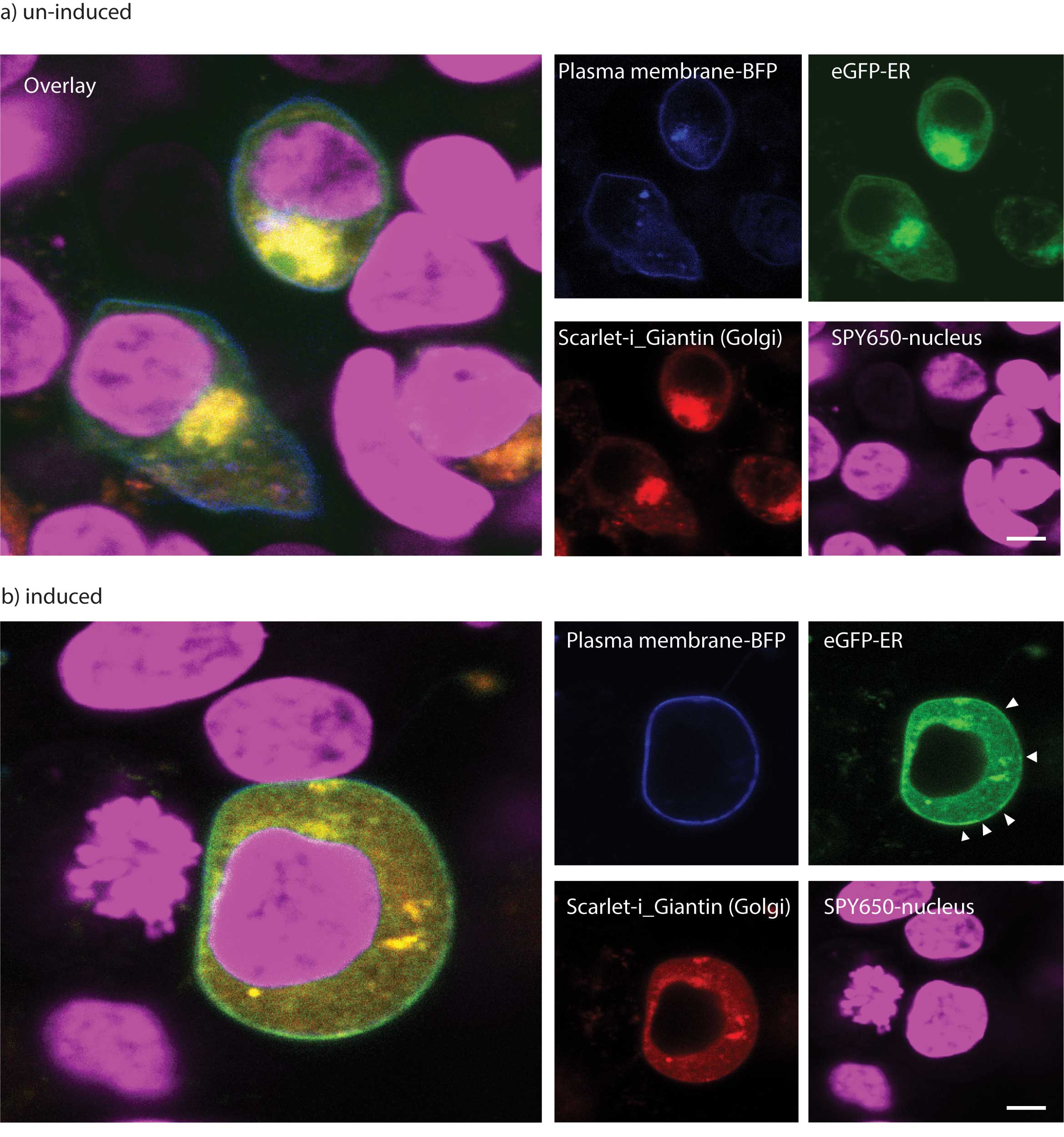
**

**Supplementary Figure 2: Colocalization of POSH Control with cell compartment markers.** HEK293T cells co-transfected with pMMZ686 (P_SV40_-Myristoylation_SS_-BFP-pA_P_PGK_-Igk-eGFP-TM-TEVcs_(3x)_-KKYL-STOP_-_pA_P_hCMV_-FLAG-NES-FRB-nTEVp-pA_P_hCMV_-FLAG-NES-FKBP-cTEVp-pA) and pmScarlet-H-Giantin-C1 were fixed before (uninduced) and after (induced) 6 h stimulation with 1 μM rapamycin. Myristoylation _Signal sequence(ss)_-BFP (blue channel) is a plasma membrane marker. eGFP is the target protein of the POSH Control system (green channel); it is mainly localized in the ER in the uninduced state and exposed on the cell surface after induction (indicated with arrows). Giantin is a marker for Golgi apparatus (red channel), and SPY650-DNA is a nuclear marker (purple channel). Scale bar, 10 μm.

**
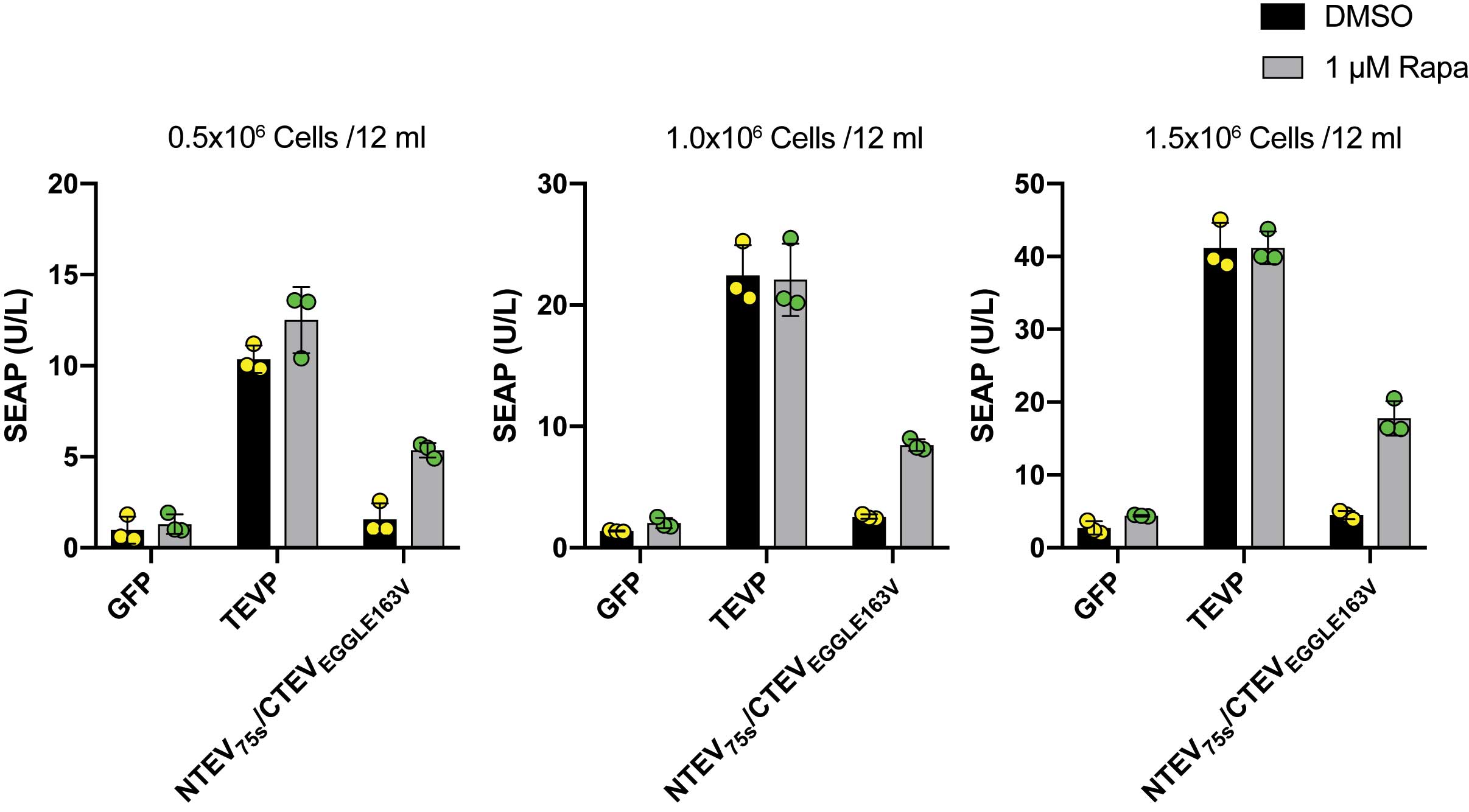
**

**Supplementary Figure 3:** **Dependency of POSH control on cell number.** HEK293T cells were plated at different cell densities (indicated above each graph) and transfected with pMMZ1363 (P_PGK_-SEAP-Furin_(3x)_-TM-TEVcs_(3x)_-KKYL-STOP_-_pA), pMMZ693 (P_hCMV_-FRB-G_4_S-cTEV_163V-EGGLE_-pA), and pMMZ 694 (P_hCMV_-FRBP-G_4_S-nTEV_75S_ -pA). Cells were induced with 1 μM rapamycin, and SEAP secreted into the medium was measured after 6 h. Bars show the mean ± s.d. of n = 3 biologically independent samples, with the individual data points. Source data are provided as a Source Data file.

**
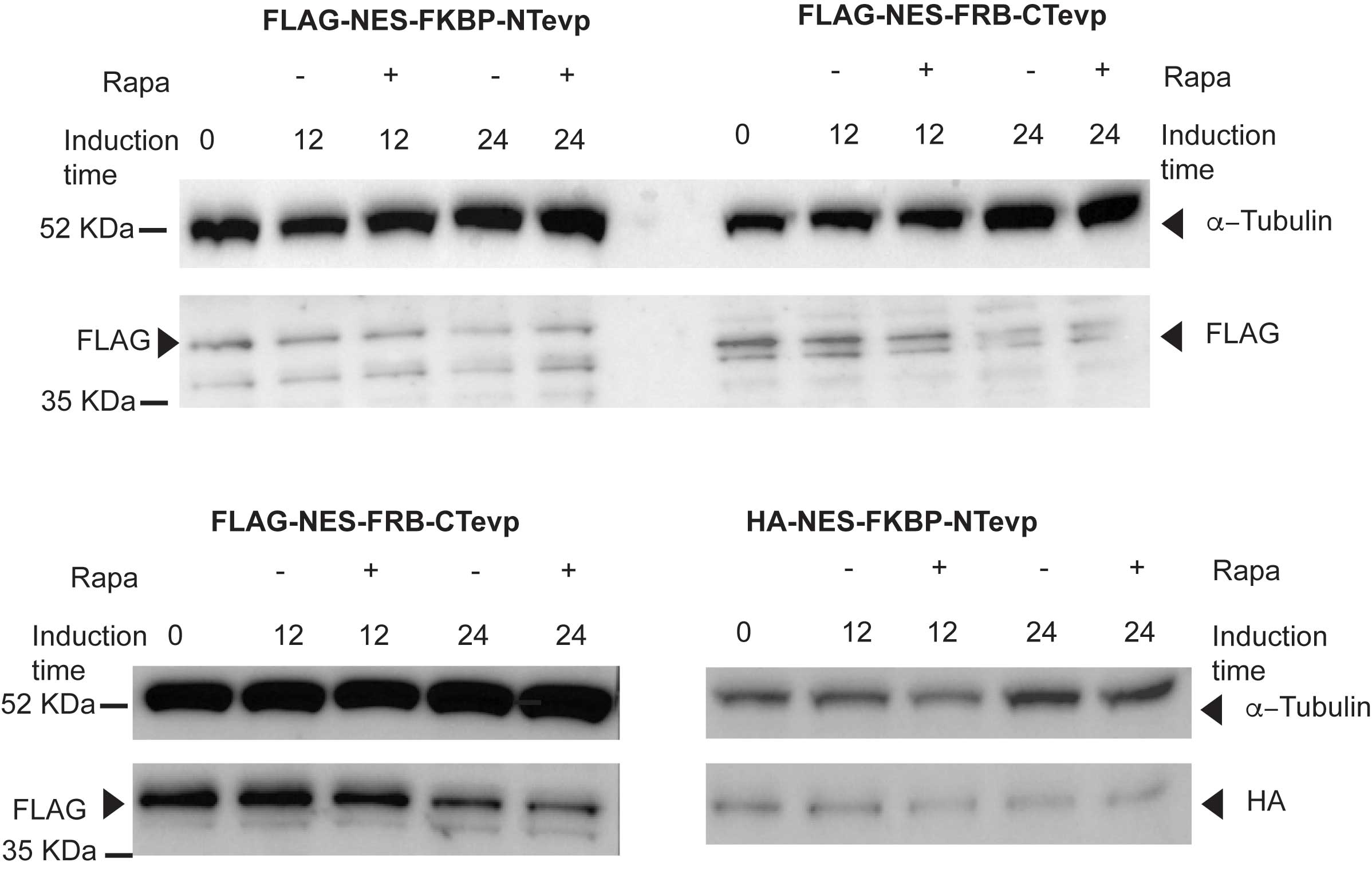
**

**Supplementary Figure 4:** **Impact of rapamycin on n/cTEVp expression.** Upper panel; HEK293T cells were co-transfected with either 50 ng of pMMZ693(P_hCMV_-FLAG_3x_-NES-FRB-G_4_S-cTEV_163V-EGGLE_-pA) and 50 ng of pFOX12 (P_hCMV_-eGFP-pA) or 50 ng of pMMZ 694 (P_hCMV_-FLAG_3x_-NES-FRBP-G_4_S-nTEV_75S_-pA) and 50 ng of pFOX12 (P_hCMV_-eGFP-pA). Cells were induced with 1 μM rapamycin for the indicated time periods, and the expression of the constructs were assessed by means of western blotting. Control cells were treated with an equimolar amount of DMSO. Lower panels; HEK293T cells were co-transfected with 50 ng of pMMZ693 (P_hCMV_-FLAG_3x_-NES-FRB-G_4_S-cTEV_163V-EGGLE_-pA) and 50 ng of pMMZ 751 (P_hCMV_-HA-NES-FRBP-G_4_S-nTEV_75S_ -pA). Cells were induced with 1 μM rapamycin for the indicated time periods, and the expression of the constructs was assessed by means of western blotting with anti-FLAG (left blot) and anti-HA (right blot). Control cells were treated with an equimolar amount of DMSO. α-Tubulin is a housekeeping gene product, used to control for total protein concentration.


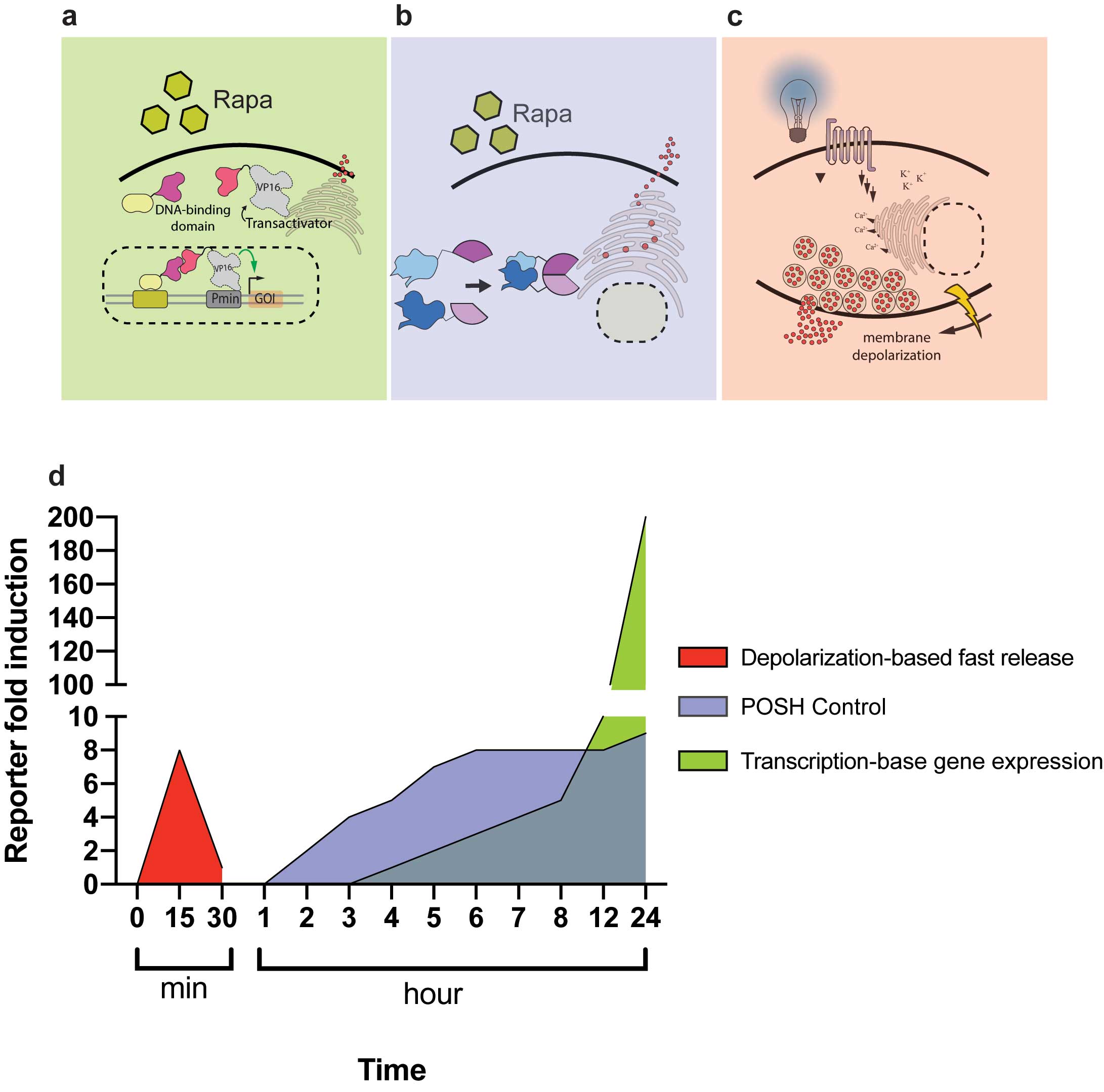


**Supplementary Figure 5:** **Comparative protein secretion kinetics.** a) Transcription-based gene expression control. HEK293T cells were co-transfected with P_CMV_-TetR-FKBP-pA, P_CMV_-FRB-VP16-pA, and pTS1017; P_TetO7-_P_CMVmin_-SEAP-pA. The cells were stimulated with 1 μM rapamycin (Rapa) and SEAP secretion was assessed every one hour for 24 h. b) POSH Control. HEK293T cells were transfected with pMMZ1363 (P_PGK_-SEAP-Furin_(3x)_-TM-TEVcs_(3x)_-KKYL-STOP_-_pA), pMMZ693 (P_hCMV_-FRB-G_4_S-cTEV_163V-EGGLE_-pA), and pMMZ 694 (P_hCMV_-FRBP-G_4_S-nTEV_75S_ -pA). Cells were induced with 1 μM rapamycin, and SEAP secreted into the medium was measured every 1 h for 24 h. c) Depolarization-mediated fast secretion. 1.1E7 β‑cells stably expressing melanopsin (pMMZ197; P_hEF‐1α_-opn4- pA) and proinsulin-nLuc (P_CMV_-Proinsulin-nLuc-pA) were illuminated with blue light (475 nm; 10 sec ON and 5 sec OFF and 300 μW/cm^2^) for 15 min and the nLuc secretion was assessed in the culture supernatant at the indicated time points for 1 h. a-c) Control cells were treated with either DMSO (a and b) or kept in the dark (c). d) The protein secretion pattern of each system over time. The fold induction of each system was calculated based on gene expression and/or protein secretion of the reporter gene/protein in the induced condition versus the uninduced condition at the indicated time points.

**
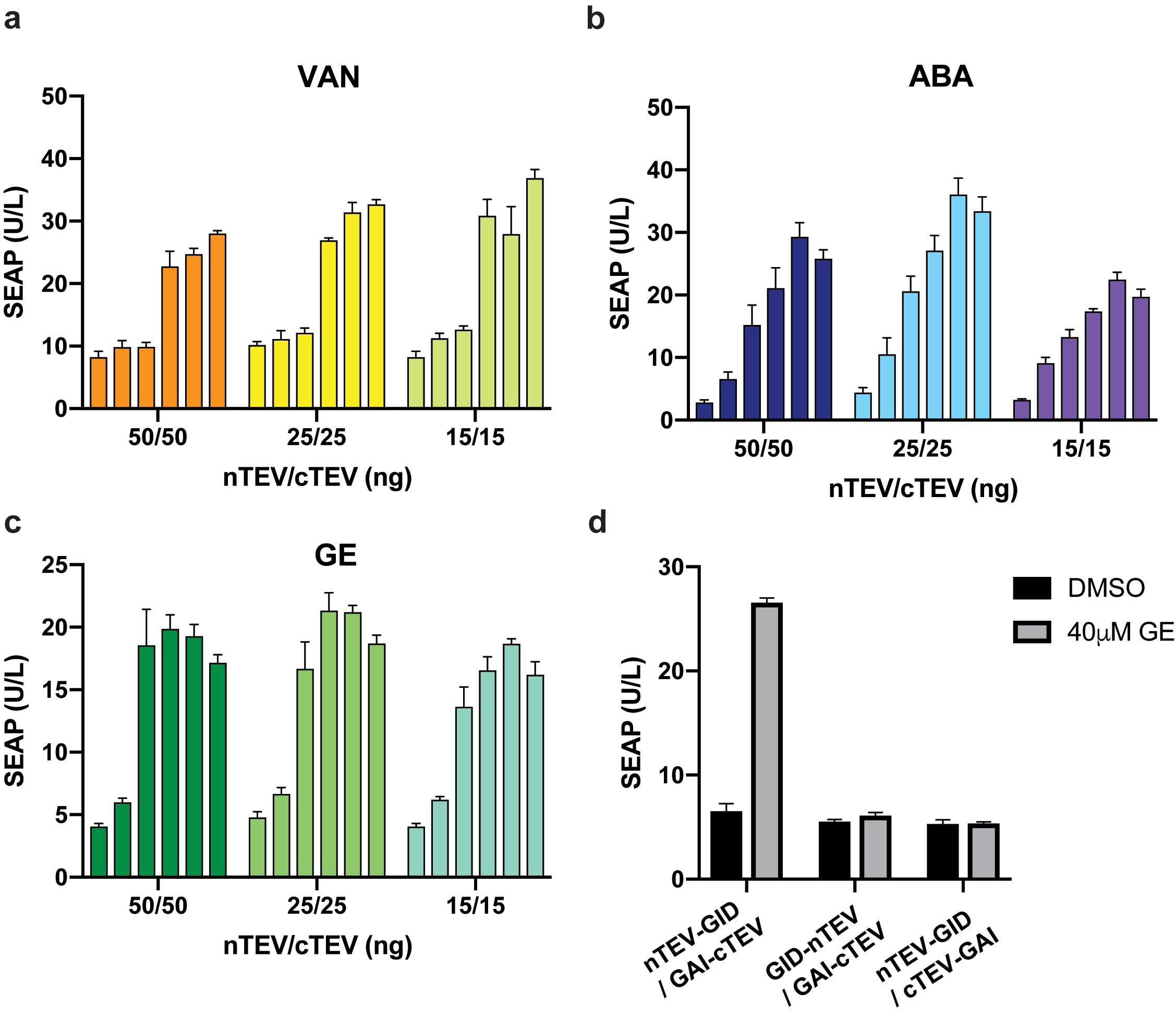
**

**Supplementary Figure 6: POSH control system employing plant-derived inducers.** HEK293T cells were transfected with the POSH control elements in varying amounts as shown, and subsequently induced with the indicated concentrations of inducers. **a**, HEK293T cells were transfected with pMMZ1363 (P_PGK_-SEAP-Furin_(3x)_-TM-TEVcs_(3x)_-KKYL-STOP_-_pA), pMMZ641 (P_hCMV_-VanR-G_4_S-nTEVp-pA), and pMMZ639 (P_hCMV_-VanR-G_4_S-cTEVp-pA), and then induced with vanillic acid. **b**, HEK293T cells were transiently co-transfected with POSH control constructs (pMMZ1363, pMMZ574; P_hCMV_-ABI-G_4_S-cTEVp-pA, and pMMZ575; P_hCMV_-PYL1-G_4_S-nTEVp-pA) and then induced with ABA. **c**, HEK293T cells were co-transfected with pMMZ1363, pMMZ637; P_hCMV_-GAI-G_4_S-cTEVp-pA, and pMMZ575; P_hCMV_-nTEVp-G_4_S-GID-pA and then induced with gibberellic acid. d) HEK293T cells were co-transfected with pMMZ1363, and the indicated combinations of pMMZ637; P_hCMV_-GAI-G_4_S-cTEVp-pA, pMMZ575; P_hCMV_-nTEVp-G_4_S-GID-pA, pMMZ648; P_hCMV_-cTEVp-G_4_S-GAI-pA, and pMMZ640; P_hCMV_-GID-G_4_S-nTEVp-pA, then induced with gibberellic acid. SEAP levels in the supernatant of cultured cells were measured after 24 h. Bars show the mean ± s.d. of n = 3 biologically independent samples. Source data are provided as a Source Data file.

**
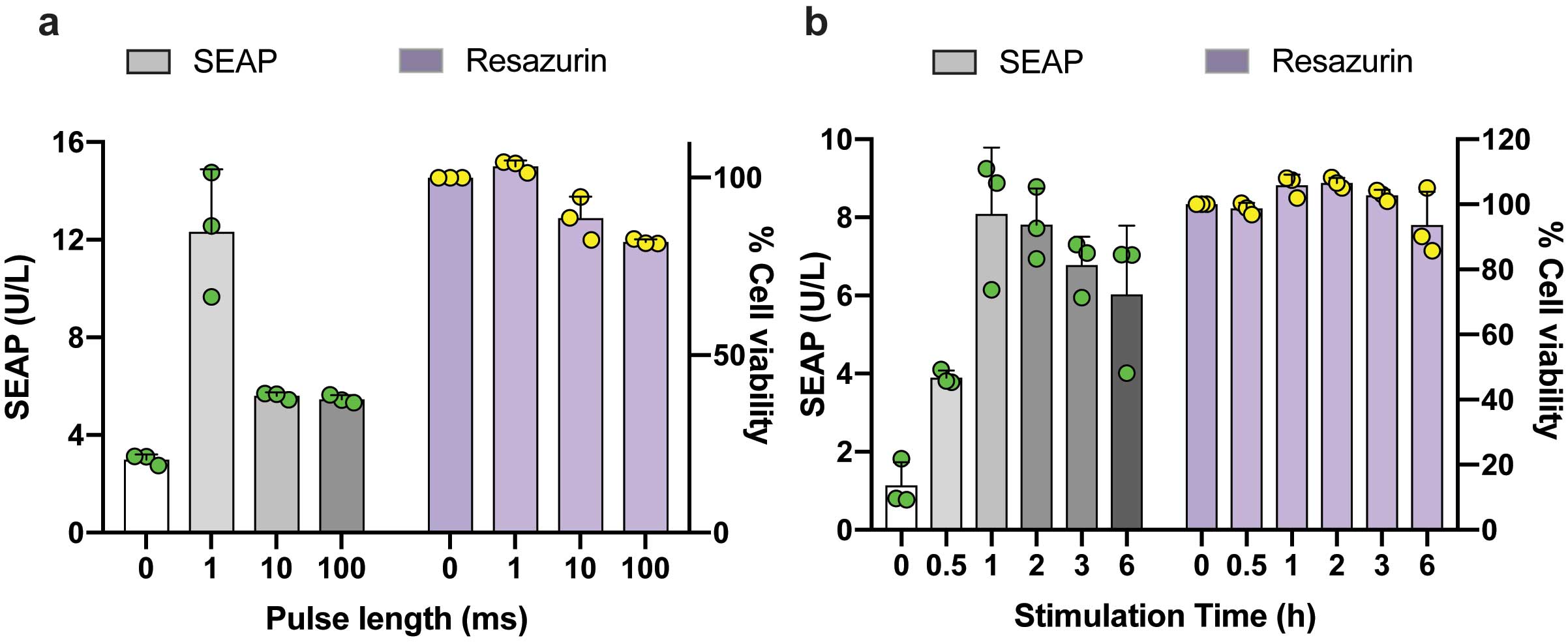
**

**Supplementary Figure 7:** **_Electro_POSH control characterization.** _electro_POSH-equipped cells were transfected with voltage-gated ion channels (pKK66; P_hEF1α_-α_1_C-P2A-K_ir_2.1-pA and pKK56; P_hEF1α_-α_2_/δ_1_-P2A-β_3_-pA) as well as the POSH calcium-sensitive controller units (pMMZ560; P_hCMV_-calmodulin-G_4_S-nTEVp-pA and pMMZ561; P_hCMV_-calmodulin binding peptide (CAMB2M)-G_4_S-cTEVp-pA), along with synthetic reporter pMMZ1363 (P_PGK_-SEAP-Furin_(3x)_-TM-TEVcs_(3x)_-KKYL-STOP_-_pA). **a** Electrical pulses of 0 – 100 ms in length were applied to the cells. The greatest inducibility was seen at 1 ms peak-to-peak (left). Viability of the electro-stimulated cells was evaluated by resazurin assay (right). **b**, Different induction times from 0 – 6 h were applied (left) to identify the optimum timeline for induction, and the viability of the electro-stimulated cells was examined by resazurin assay (right). Bars show the mean ± s.d. of n = 3 biologically independent samples, with the individual data points. Source data are provided as a Source Data file.


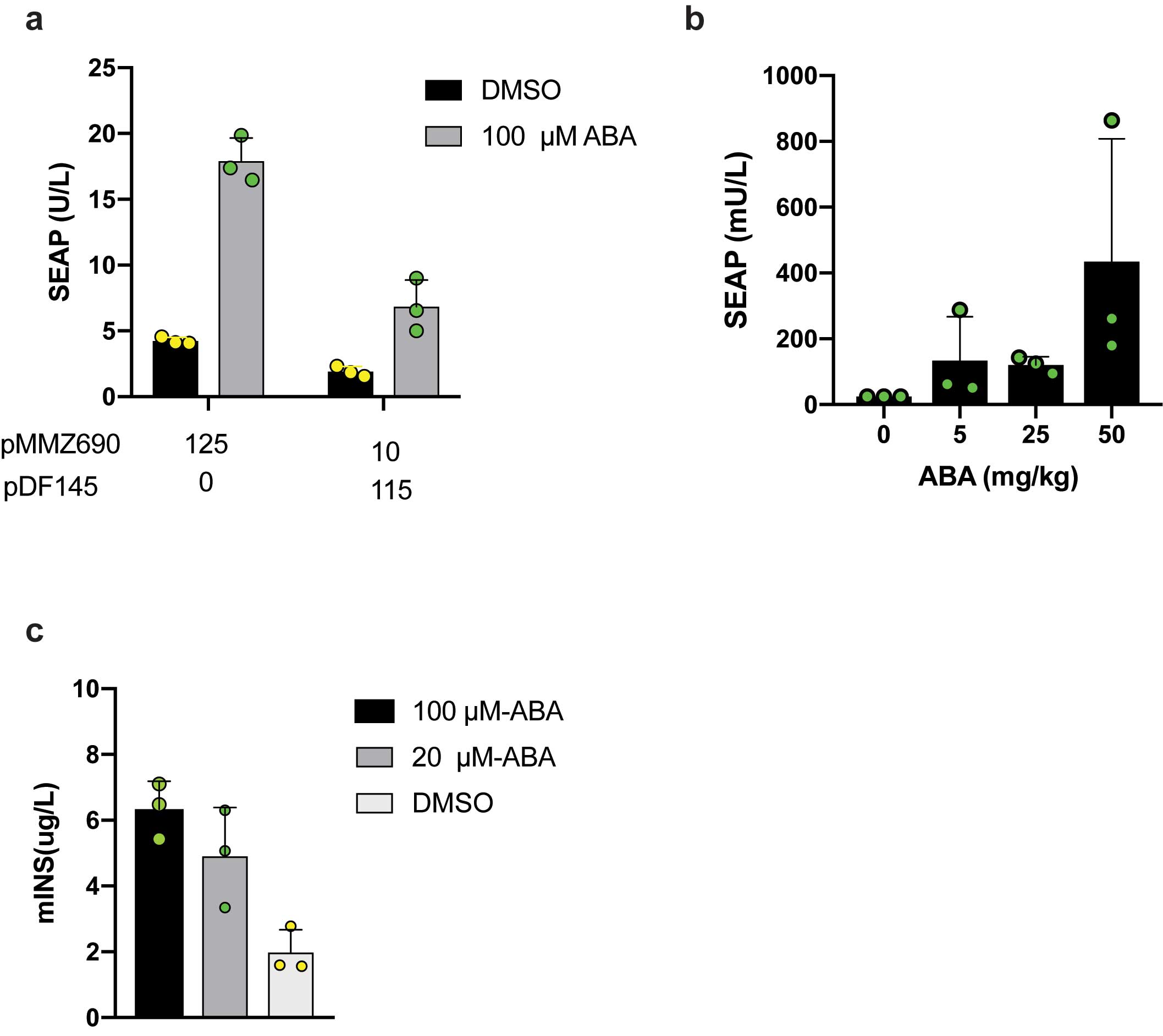


**Supplementary Figure 8:** **Application of all-in-one POSH control in vitro and in vivo. a**, HEK293T cells were transfected with POSH control constructs pMMZ690 (P_PGK_-SEAP-Furin_(3x)_-TM-TEVcs_(3x)_-KKYL-STOP_-_pA_P_hCMV_-ABI-G_4_S-cTEVp-pA_pMMZ575; P_hCMV_-PYL1-G_4_S-nTEVp-pA) and induced with 50 μM abscisic acid (ABA). **b**, Mice (n = 3) were hydro-injected with pMMZ690 through a tail vein and SEAP levels in blood were measured 24 h after induction with different concentrations of abscisic acid (ABA). Bars show the mean ± s.d. (n = 5), with the individual data points. The statistical significance of differences between indicated groups was calculated using a two-tailed, unpaired Student’s t-test. Source data are provided as a Source Data file. **c**, POSH-control of insulin secretion. HEK293T cells were transfected with POSH control constructs (pMMZ635; P_PGK_-mInsulin-Furin-TM-TEVcs_(3x)_-KKYL-STOP_-_pA), pMMZ574; P_hCMV_-ABI-G_4_S-cTEVp-pA, and pMMZ575; P_hCMV_-PYL1-G_4_S-nTEVp-pA) and induced with the indicated concentration of abscisic acid (ABA). Insulin levels in the supernatant of cultured cells were measured at 24 h after induction. Bars show the mean ± s.d. of n = 3 biologically independent samples, with the individual data points. Source data are provided as a Source Data file.

**Supplementary Table 1**. Plasmids used and designed in this study.

| Plasmid | Description and cloning strategy | Reference |
| --- | --- | --- |
| pMMZ686 | P_SV40_-Myristoylation_SS_-BFP-pA_P_PGK_-Igk-eGFP-TM-TEVcs_(3x)_-KKYL-STOP_-_pA_P_hCMV_-FLAG-NES-FRB-nTEVp-pA_P_hCMV_-FLAG-NES-FKBP-cTEVp-pA  Mammalian expression vector encoding plasma membrane marker (Myr-BFP), synthetic reporter expressing SEAP and protease-based controller units (FRB-nTEV/FKBP-cTEV).  P_SV40_-Myristoylation_SS_-BFP-pA was amplified with oMMZ405/oMMZ406, P_PGK_-Igk-eGFP-TM-TEVcs_(3x)_-KKYL-STOP_-_pA was amplified with oMMZ407/oMMZ408, P_hCMV_-FLAG-NES-FRB-nTEVp-pA was amplified with oMMZ 409/ oMMZ 410 and P_hCMV_-FLAG-NES-FKBP-cTEVp-pA was amplified with oMMZ 411/oMMZ412. Gibson assembly reaction was applied to the fragments to create a single plasmid expressing the four cassettes^1^. | This work |
| pMMZ623 | P_SV40_-Myristoylation_SS_-BFP-pA  Mammalian expression vector encoding BFP fused N-terminally to a plasma membrane anchoring domain (myristoylation). BFP was digested with SpeI/HindIII and cloned into pVH017 plasmid restricted with NheI/HindIII. | This work |
| pMMZ622 | P_PGK_-Igk-eGFP-TM-TEVcs_(3x)_-KKYL-STOP_-_pA  Mammalian expression vector encoding eGFP fused to TM, TEVcs and KKYL. The plasmid was created by Gibson assembly through amplification of Igk-eGFP from pMMZ695 and P_PGK_-TM-TEVcs_(3x)-_KKYL-STOP-pA. | This work |
| pMMZ595 | P_PGK_-SEAP-TM-TEVcs_(3x)-_KKYL-STOP-pA  Synthetic reporter cassette without the furin site. | This work |
| pFOX12 | P_hCMV_-eGFP-pA  Mammalian expression plasmid encoding eGFP. | This work |
| pMMZ695 | P_hCMV_-Igk-eGFP-pA  Mammalian expression plasmid encoding secreted eGFP. | This work |
| pMMZ1363 | P_PGK_-SEAP-Furin_(3x)_-TM-TEVcs_(3x)_-KKYL-STOP_-_pA  Mammalian expression vector encoding SEAP-Furin_(3x)_-TM-TEVcs_(3x)_-KKYL. | This work |
| pMMZ687 | P_hCMV_- FLAG_3x_-NES-FKBP-G_4_S-nTEVp-pA  Mammalian expression vector encoding the FKBP-nTEVp (addgene ID; 311961). | ^2^ |
| pMMZ688 | P_hCMV_- FLAG_3x_-NES-FRB-G_4_S-cTEVp-pA  Mammalian expression vector encoding the FRB-cTEVp (addgene ID; 311962). | ^2^ |
| pMMZ507 | P_hCMV_-nTEVp-G_4_S-FRB-(G_4_S)_3x_-FKBP-G_4_S-cTEVp_-_pA  Mammalian expression vector encoding the POSH control elements all-in-one.  This plasmid was made through serial cloning of nTEV-G4S-FRB, (G4S), and FKBP-G4S-cTEVp through compatible cohesive ends. | This work |
| pMMZ691 | P_hCMV_- FLAG_3x_-NES-FRB-G_4_S-cTEVp_(EGGLE)_-pA  Mammalian expression vector encoding FRB-cTEVp. cTEVp carries an EGGLE tail in the C-terminus.  EGGLE was inserted through site-directed mutagenesis using oMMZ272/oMMZ273 in pMMZ688 plasmid. | This work |
| pMMZ692 | P_hCMV_- FLAG_3x_-NES-FRB-G_4_S-cTEVp_(163V)_-pA  Mammalian expression vector encoding FRB-cTEVp. cTEVp carries mutation 163V.  163V was inserted through site-directed mutagenesis using oMMZ219/oMMZ220 in pMMZ688 plasmid. | This work |
| pMMZ693 | P_hCMV_- FLAG_3x_-NES-FRB-G_4_S-cTEVp_(EGGLE-163V)_-pA  Mammalian expression vector encoding FRB-cTEVp. cTEVp carries both an EGGLE tail at the C-terminus and 163V.  163V was inserted through site-directed mutagenesis using oMMZ219/oMMZ220 in pMMZ691 plasmid. | This work |
| pMMZ694 | P_hCMV_- FLAG_3x_-NES-FKBP-G_4_S-nTEVp_(75S)_-pA  Mammalian expression vector encoding FKBP-nTEVp. nTEVp carries mutation 75S.  75S was inserted through site-directed mutagenesis using oMMZ221/oMMZ222 in pMMZ687 plasmid. | This work |
| pMMZ565 | P_PGK_-SEAP-Furin_(3x)_-TM-SbMVcs_(3x)_-KKYL-STOP_-_pA  Synthetic reporter unit encoding SEAP with a recognition site for SbMV protease.  SbMVcs was amplified by oMMZ270/oMMZ271 and cloned into pMMZ1363 through Gibson assembly | This work |
| pMMZ696 | P_hCMV_-FKBP-G_4_S-cSbMVp-pA  Mammalian expression vector encoding the FKBP-cSbMVp (addgene ID; 232050). | ^3^ |
| pMMZ697 | P_hCMV_-FRB-G_4_S-nSbMVp-pA  Mammalian expression vector encoding the FKBP-nSbMVp (addgene ID; 232046). | ^3^ |
| pMMZ641 | P_hCMV_- FLAG_3x_-NES-VanR-G_4_S-nTEVp -pA  Mammalian TEVp-based controller unit containing vanillic acid-responsive (VanR) elements. VanR was amplified by oMMZ452/oMMZ453 and cloned into pMMZ694 oMMZ39/oMMZ240. | This work |
| pMMZ639 | P_hCMV_- FLAG_3x_-NES-VanR-G_4_S-cTEVp -pA  Mammalian TEVp-based controller unit containing vanillic acid-responsive (VanR) elements. VanR was amplified by oMMZ452/oMMZ453 and cloned into pMMZ693 using primers oMMZ239/240. | This work |
| pMMZ574 | P_hCMV_- FLAG_3x_-NES-ABI-G_4_S-cTEVp -pA  Mammalian TEVp-based controller unit containing ABA-responsive (ABI) elements. ABI was amplified by oMMZ310/oMMZ311 and cloned into pMMZ693 using primers oMMZ239/240. | This work |
| pMMZ575 | P_hCMV_- FLAG_3x_-NES-PYL1-G_4_S-nTEVp -pA  Mammalian TEVp-based controller unit containing ABA-responsive (PYL1) elements. pYL1 was amplified by oMMZ312/oMMZ313 and cloned into pMMZ694 using primers oMMZ39/oMMZ240. | This work |
| pMMZ637 | P_hCMV_-GAI-G_4_S-cTEVp -pA  Mammalian TEVp-based controller unit containing GA-responsive (GAI) elements. | This work |
| pMMZ666 | P_hCMV_-nTEVp-G_4_S-GID-pA  Mammalian TEVp-based controller unit containing GA-responsive (GID) element in the C-terminus. | This work |
| pMMZ640 | P_hCMV_-GID-G_4_S-nTEVp -pA  Mammalian TEVp-based controller unit containing GA-responsive (GID) element in the N-terminus. | This work |
| pMMZ564c | P_hCMV_-pMag-(G_4_S)_3x_-nTEVp -pA  Blue-light-responsive domain (pMag) fused to protease unit (nTEVp). | This work |
| pMMZ564b | P_hCMV_-nHighMag-(G_4_S)_3x_-cTEVp -pA  Blue-light-responsive domain (nHighMag) fused to protease unit (cTEVp). | This work |
| pMMZ564bn | P_hCMV_-cTEVp -(G_4_S)_3x_-nHighMag-pA  Blue-light responsive domain (nHighMag) is C-terminally fused to protease unit (cTEVp). | This work |
| pMMZ678 | P_hCMV_-nTEVp-(G_4_S)_2x_-nHighMag-pA  Blue-light-responsive domain (nHighMag) is C-terminally fused to protease unit (nTEVp) with two repeats of G4S linker. | This work |
| pMMZ662 | P_hCMV_-pMag-(G_4_S)_3x_-cTEVp -pA  Blue-light-responsive domain (pMag) is fused to protease unit (cTEVp). | This work |
| pMMZ670 | P_hCMV_-cTEVp -(G_4_S)_3x_-pMag-pA  Blue-light-responsive domain (pMag) is fused to protease unit (cTEVp) N-terminally. | This work |
| pKK66 | ITR-P_hEF1α_-α1C-P2A-K_ir_2.1-pA-ITR:PRPBSA-BFP-P2A-PuroR-pA-ITR  Mammalian expression plasmid encoding voltage-gated ion channels. | ^4^ |
| pKK56 | P_hEF1α_-α_2_/δ_1_-P2A-β_3_-pA  Mammalian expression plasmid encoding α2/δ1 and β3. | ^4^ |
| pMMZ560 | P_hCMV_-Calmodulin (CAL)-G_4_S-nTEVp-pA  Mammalian expression plasmid encoding calcium-sensitive domain (CAL)^5^ fused to nTEVp. Calmodulin was amplified by oMMZ241/242 and cloned to pMMZ694 using primers oMMZ39/oMMZ240. | This work |
| pMMZ561 | P_hCMV_-Calmodulin-binding peptide (CAMB2M)-G_4_S-cTEVp-pA  Mammalian expression plasmid encoding calcium-sensitive domain (CAMB2M)^5^ fused to cTEVp. CAMB2M was amplified by oMMZ243/oMMZ244 and cloned into pMMZ693 using primers oMMZ239/240. | This work |
| pMM690 | P_PGK_-SEAP-Furin_(3x)_-TM-TEVcs_(3x)_-KKYL-STOP_-_pA_ P_hCMV_- FLAG_3x_-NES-ABI-G_4_S-cTEVp-pA_P_hCMV_- FLAG_3x_-NES-PYL1-G_4_S-nTEVp -pA  Mammalian expression vector encoding synthetic reporter expressing SEAP and protease-based controller units (PYL1-nTEV/ABI-cTEV).  P_PGK_- SEAP-Furin_(3x)_-TM-TEVcs_(3x)_-KKYL-STOP_-_pA was amplified with oMMZ405/oMMZ408 from pMMZ1368, P_hCMV_- FLAG_3x_-NES-ABI-G_4_S-cTEVp-pA was amplified with oMMZ 409/ oMMZ 410 from pMMZ574 and P_hCMV_- FLAG_3x_-NES-PYL1-G_4_S-nTEVp -pA was amplified with oMMZ 411/oMMZ412 from pMMZ575. Gibson assembly reaction was applied to the fragments to create a single plasmid expressing the three cassettes. | This work |
| pMM689 | P_PGK_-insulin-Furin-TM-TEVcs_(3x)_-KKYL-STOP_-_pA_ P_hCMV_- FLAG_3x_-NES-ABI-G_4_S-cTEVp-pA_P_hCMV_- FLAG_3x_-NES-PYL1-G_4_S-nTEVp -pA  Mammalian expression vector encoding synthetic reporter expressing mouse insulin (mInsulin) and protease-based controller units (PYL1-nTEV/ABI-cTEV).  P_PGK_-mInsulin-Furin-TM-TEVcs_(3x)_-KKYL-STOP_-_pA was amplified with oMMZ405/oMMZ408 from pMMZ635, P_hCMV_- FLAG_3x_-NES-ABI-G_4_S-cTEVp-pA was amplified with oMMZ 409/ oMMZ 410 from pMMZ574 and P_hCMV_- FLAG_3x_-NES-PYL1-G_4_S-nTEVp -pA was amplified with oMMZ 411/oMMZ412 from pMMZ575. Gibson assembly reaction was applied to the fragments to create a single plasmid expressing the three cassettes. | This work |
| pMM635 | P_PGK_-mInsulin-Furin-TM-TEVcs_(3x)_-KKYL-STOP_-_pA  mInsulin-Furin was amplified by oMMZ422/oMMZ423 and cloned into pMMZ1363 backbone using primers oMMZ424/oMMZ425 | This work |
| pDF145 | Bacterial RNA production vector without mammalian promoter activity (P_T7_-SpAH-Env140ac). | Fuchs et al.^6^ |
| Pm-Scarlet-H-Giantin-C1 | P_hCMV_ Pm-Scarlet-H-Giantin-C1-pA  Expressing human giantin (Golgi apparatus biomarker) fused to scarlet fluorescent protein (addgene#; 85049) | Bindels et al. ^7^ |
| pMMZ751 | P_hCMV_- HA-NES-FKBP-G_4_S-nTEVp_(75S)_-pA  Mammalian expression vector encoding FKBP-nTEVp. nTEVp This construct is N-terminally tagged to a HA epitope (YPYDVPDYA). | This work |

**Supplementary Table 2**. Oligonucleotides utilized for PCR in this study.

|  | Name | Sequence |
| --- | --- | --- |
| 1 | oMMZ405 | AACGCTCTATGGTCTAAAGATTTAAATcACGCGTGGTACCCTCGAG |
| 2 | oMMZ406 | AAACGTGCAATAGTATCCAGTTTATTTAAATTctagagccccagctggttctttc |
| 3 | oMMZ407 | AAACTGGATACTATTGCACGTTTAAATcACGCGTGGTACCCTCGAG |
| 4 | oMMZ408 | AAACATCAGGCATCATTAGGTTTATTTAAATTctagagccccagctggttctttc |
| 5 | oMMZ409 | AAACCTAATGATGCCTGATGTTTAAATGCcagatatacgcgttgacattg |
| 6 | oMMZ410 | AAACTAAGCTATGTGAACCGTTTATTTAAATTctagagccccagctggttctttc |
| 7 | oMMZ411 | AAACGGTTCACATAGCTTAGTTTAAATCCAGATATACGCGTTGACATTGATTATTGACTAG |
| 8 | oMMZ412 | AACCCCGATTGAGATATAGATTTATTTAAATTctagagccccagctggttctttc |
| 9 | oMMZ272 | cctggaaTAAGCGGCCGCTCGAGTC |
| 10 | oMMZ273 | cccccttcATTCATGAGTTGAGTCGCTTCCTTAAC |
| 11 | oMMZ219 | AGATGGGTTCgttGTTGGTATAC |
| 12 | oMMZ220 | CTAGTTGATACTAATGGACTG |
| 13 | oMMZ221 | TTTGCAACAAagcCTCATTGATGGGAG |
| 14 | oMMZ222 | GTCGTGGTGTTCTTGACC |
| 15 | oMMZ270 | cttttgtcaggttcaggggaaAGTGTTAGCCTCcagtcaggaagtggtGAGAGCGTGAGTCTGcagagtggatcaggcgagAGTGTGAGTCTTcaaag |
| 16 | oMMZ271 | gaatagggccctctagatgcatgttacagatatttcttaccgctgccgctttgAAGACTCACACTctc |
| 17 | oMMZ452 | gctctggccagctcctttagcgtgtcctccggaATGGACATGCCGCGCATAAAG |
| 18 | oMMZ453 | agacaagtcagagactgagtcGGATCCGTCGGCGCGAATGCTCCACG |
| 19 | oMMZ310 | agcgtgtcctccggaATGACGCGTGTGCCTTTG |
| 20 | oMMZ311 | gacccgccaccaccggatccCTTCAAATCAACCACCACCAC |
| 21 | oMMZ239 | tccggaggacacgctaaaggag |
| 22 | oMMZ240 | ggatccggtggtggcgggtc |
| 23 | oMMZ312 | CTCCGTTTCTTCAGGGATGggcgcgccaactcaagac |
| 24 | oMMZ313 | CACCGCCGCCACTCCCgttcatagcttcagtgatcg |
| 25 | oMMZ239 | tccggaggacacgctaaaggag |
| 26 | oMMZ240 | ggatccggtggtggcgggtc |
| 27 | oMMZ241 | ctcctttagcgtgtcctccggaatggaccaactgactgaagag |
| 28 | oMMZ242 | gacccgccaccaccggatccagtgctgtccaggcccagcagag |
| 29 | oMMZ243 | ctcctttagcgtgtcctccggattcaacgctcgcaggaagctg |
| 30 | oMMZ244 | gacccgccaccaccggatccagtgctgtccaggcccagcag |
| 31 | oMMZ422 | GCGgaattcgccaccatgGCCCTGTGGATG |
| 32 | oMMZ423 | agagcctgaGGATCCGCTAGctctcttgtatc |
| 33 | oMMZ424 | ggtggcgaattcCGCTTC |
| 34 | oMMZ425 | GGATCCtcaggctctggtattattatg |

**Abbreviations**

**P_hCMV_**, human cytomegalovirus immediate early promoter; **P_SV40_**, simian virus 40 promoter; **P_PGK_**, phosphoglycerate kinase promoter; **P_RPBSA_,** constitutive synthetic mammalian promoter; **SEAP**, human placental secreted alkaline phosphatase; **mINS**, mouse insulin; **eGFP,** enhanced green fluorescent protein; **BFP**, blue fluorescent protein; **PuroR,** puromycin resistance gene; **pA,** polyadenylation signal, **SS**, signal sequence; **PCR**, polymerase chain reaction; **G_4_S**, Gly-Gly-Gly-Gly-Ser; **Myr_ss_**, myristoylation signal sequence; **PFA**, paraformaldehyde. **IgK**, secretion signal peptide; **FRB,** FKBP-rapamycin binding protein; **FKBP**, FK506 binding protein; **NES**, nuclear export signal; **TEVp**, tobacco etch virus protease; **SbMVp**, soybean mosaic virus protease; **TM**, transmembrane domain; **VanR**, transmembrane domain; **VanR**, vanillic acid-responsive transcriptional repressor; **CAL**, calmodulin; **CAMB2M**, calmodulin-binding peptide.

**References**

1. Weissmann, F. *et al.* biGBac enables rapid gene assembly for the expression of large multisubunit protein complexes. *Proc. Natl. Acad. Sci.* (2016). doi:10.1073/pnas.1604935113

2. Dolberg, T. B. *et al.* Computation-guided optimization of split protein systems. *Nat. Chem. Biol. 2021 175* **17**, 531–539 (2021).

3. Fink, T. *et al.* Design of fast proteolysis-based signaling and logic circuits in mammalian cells. *Nat. Chem. Biol. 2018 152* **15**, 115–122 (2018).

4. Krawczyk, K. *et al.* Electrogenetic cellular insulin release for real-time glycemic control in type 1 diabetic mice. **368**, 993–1001 (2020).

5. Wang, W. *et al.* A light- and calcium-gated transcription factor for imaging and manipulating activated neurons. *Nat. Biotechnol. 2017 359* **35**, 864–871 (2017).

6. Ausï Ander, S., Fuchs, D., Urlemann, S. H. ¨, Ausï Ander, D. & Fussenegger, M. Engineering a ribozyme cleavage-induced split fluorescent aptamer complementation assay. *Nucleic Acids Res.* **44**, 94 (2016).

7. Bindels, D. S. *et al.* mScarlet: a bright monomeric red fluorescent protein for cellular imaging. *Nat. Methods* **14**, 53–56 (2017).
